# HumanMycobiomeScan: a new bioinformatics tool for the characterization of the fungal fraction in metagenomic samples

**DOI:** 10.1186/s12864-019-5883-y

**Published:** 2019-06-15

**Authors:** Matteo Soverini, Silvia Turroni, Elena Biagi, Patrizia Brigidi, Marco Candela, Simone Rampelli

**Affiliations:** Department of Pharmacy and Biotechnology, Unit of Microbial Ecology of Health, University of Bologna, Via Belmeloro 6, 40126 Bologna, Italy

**Keywords:** Mycobiome, Microbiome, Metagenomics

## Abstract

**Background:**

Modern metagenomic analysis of complex microbial communities produces large amounts of sequence data containing information on the microbiome in terms of bacterial, archaeal, viral and eukaryotic composition. The bioinformatics tools available are mainly devoted to profiling the bacterial and viral fractions and only a few software packages consider fungi. As the human fungal microbiome (human mycobiome) can play an important role in the onset and progression of diseases, a comprehensive description of host-microbiota interactions cannot ignore this component.

**Results:**

HumanMycobiomeScan is a bioinformatics tool for the taxonomic profiling of the mycobiome directly from raw data of next-generation sequencing. The tool uses hierarchical databases of fungi in order to unambiguously assign reads to fungal species more accurately and > 10,000 times faster than other comparable approaches. HumanMycobiomeScan was validated using in silico generated synthetic communities and then applied to metagenomic data, to characterize the intestinal fungal components in subjects adhering to different subsistence strategies.

**Conclusions:**

Although blind to unknown species, HumanMycobiomeScan allows the characterization of the fungal fraction of complex microbial ecosystems with good performance in terms of sample denoising from reads belonging to other microorganisms. HumanMycobiomeScan is most appropriate for well-studied microbiomes, for which most of the fungal species have been fully sequenced. This released version is functionally implemented to work with human-associated microbiota samples. In combination with other microbial profiling tools, HumanMycobiomeScan is a frugal and efficient tool for comprehensive characterization of microbial ecosystems through shotgun metagenomics sequencing.

**Electronic supplementary material:**

The online version of this article (10.1186/s12864-019-5883-y) contains supplementary material, which is available to authorized users.

## Background

We generally use the term ‘human holobiont’ to refer to human beings and their microbiome, as in the bacterial component, but the microbial communities that inhabit our bodies also include other components, such as fungi and viruses [1]. In particular, fungi have been reported to contribute less than 1% to the human gut microbiome [2]; however, it is likely that this figure underestimates their relevance to human health [3]. Alterations in the fungal fraction of the gut microbial ecosystem have indeed been observed in inflammatory bowel disease and immunocompromised patients [4, 5], suggesting that the mycobiome (i.e. the fungal microbiome) may act as a reservoir of potential opportunistic pathogens or pathobionts, in particular in conditions of vulnerability [6, 7]. Moreover, fungi should also be regarded as a common component of the microbiome, as demonstrated by the regular detection of *Saccharomyces*, *Malassezia* and *Candida* species in our gastrointestinal tract [8]. Like other microbiota components fungi can as well establish an intense cross-talk with the host immune system, thus having potential health beneficial and probiotic effects [9]. For all these reasons, profiling the taxonomic structure of fungal communities is important to explore their role in the biology of the human holobiont, but also to pave the way for new surveillance strategies and new opportunities to disentangle complex disorders and other complications [4, 5].

The characterization of the mycobiome structure can be done using both culture-dependent and independent methods [4]. Culture-dependent techniques, which generally combine methods such as microscopy [10], biochemical assays [11] and growth on selective media [12], represent a classical approach for the profiling of complex microbial ecosystems, and have the great advantage of allowing the determination of the viable fraction of the mycobiome. However, this is a time-consuming approach and, most importantly, blind to species that are obligate symbionts or have complex nutritional requirements or that are otherwise hard or impossible to raise in culture [13]. On the other hand, culture-independent methods basically rely on the amplification and sequencing of ITS (Internal Transcribed Spacer) or 18S rDNA phylogenetic markers [4], or on multi-gene metabarcoding [14], followed by dedicated bioinformatics pipelines for the inference of the community structure, such as QIIME [15, 16], CloVR-ITS [17], UPARSE [18], CONSTAX [19] and MICCA [20]. However, no gold standard approach for culture-independent mycobiome analysis has yet been developed, as highlighted by the variety of genomic regions and techniques used in different studies [2, 5, 21–23]. In this context, a pipeline specifically devoted to the characterization of the mycobiome based on metagenomic reads from whole genome sequencing of microbial communities is completely missing. In an attempt to bridge this gap, here we present HumanMycobiomeScan, a new bioinformatics tool that taxonomically profiles the mycobiome within the original microbiome, requiring only a few minutes to process thousands of metagenomics reads. HumanMycobiomeScan works with shotgun reads to detect traces of fungal DNA and estimate the abundance profiles by filtering out human and bacterial sequences and mapping the remaining sequences onto a hierarchical fungal database. HumanMycobiomeScan is available at the website: http://sourceforge.net/projects/hmscan.

## Implementation

### Workflow of the software

**Fig. 1:**
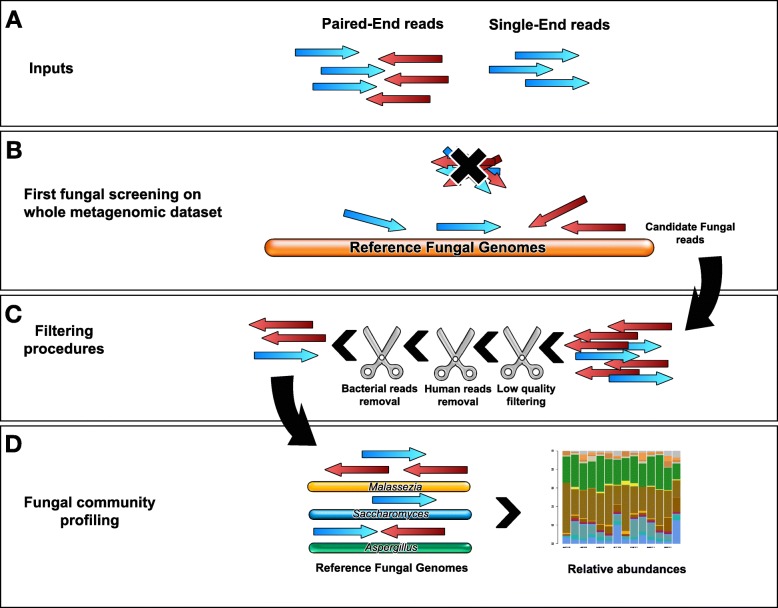
Analysis workflow of HumanMycobiomeScan. **a** Inputs are single- (.fastq) or paired-end (.fastq or compressed .fastq) reads. **b** Candidate fungal reads are screened by mapping onto reference fungal genomes contained in a precompiled database. This allows for a first reduction of the sample size, lowering the number of sequences that will be subjected to further steps. **c** Three filtration steps are carried out to eliminate low quality reads as well as reads belonging to humans and bacteria. **d** The remaining sequences are realigned onto the fungal genome database for definitive taxonomic assignment of the reads. The results are tabulated as both abundance profiles and read counts, and represented by bar plots

HumanMycobiomeScan directly analyzes metagenomics reads to detect and extract fungal sequences without any pre-processing steps. Accepted input files are single- or paired-end reads in .fastq format [24] (.bzip2, .gzip and .zip compressions are accepted as well) produced by shotgun sequencing. The HumanMycobiomeScan database is based on the complete fungal genomes available at the NCBI website (downloaded in February 2018) [25]. The NCBI IDs for each entry included in the database are reported in Additional file 1, together with the reference size (for downstream normalization purposes). The database contains a total of 1213 entries, corresponding to 66 different fungal genomes (referred to as Fungi_LITE on the project website). A second database containing 38,000 entries (including “not completed” genome records), corresponding to 265 different fungal genomes, is available for download (referred to as Fungi_FULL), and can be obtained and formatted by following the instructions on the project web page (https://sourceforge.net/projects/hmscan/). See Additional file 2 for the phylum-level assignment of the fungal genomes within the two databases. The schematic workflow of HumanMycobiomeScan is reported in Fig. 1. In detail, metagenomic reads are aligned to the fungal genome database using bowtie2 [26]. This first step is necessary to identify candidate fungal reads and reduce the sample size by filtering out sequences that do not match the reference database. It is important to note that performing this procedure at the beginning of the analysis allows for a significant decrease (~100X) in the time required for the subsequent parts of the pipeline. Afterwards, a quality-filtering step of putative fungal reads was implemented by modifying the processing procedure of the Human Microbiome Project (HMP) [27]. Briefly, sequences are trimmed for low quality scores (less than 3) using a modified version of the script trimBWAstyle.pl directly on BAM files [28]. Additionally, reads shorter than 60 bases are discarded. Since the input sequences may derive from human-associated samples, such as feces or tissues, it is plausible to expect a certain amount of contamination due to human and bacterial sequences. To remove these contaminations as accurately as possible, a double filtering step is performed using BMTagger [29]. BMTagger is a proficient tool capable of discriminating between human or bacterial and other reads by comparing short fragments of 18 bases (18 mers) originated from both the input sequences and the reference human or bacterial database. Specifically, we used the hg19 database for human sequences [30] and a custom bacterial database, also used for ViromeScan, including bacteria from human specimens and the archaeon that normally inhabits the human body and especially the intestine, i.e. *Methanobrevibacter smithii* [31]. The released version of HumanMycobiomeScan is thus functionally implemented to work with human-associated microbiota samples. Nevertheless, the databases can be customized by the user, making the program flexible and capable of working with datasets of various origin (e.g. mycobiomes associated with soil, water, air or other animals). As a final step of the workflow, filtered reads are matched again to the fungal database using bowtie2 [26] for definitive taxonomic assignment. The taxonomic affiliation is deduced by matching the result of the taxonomic assignment with an annotated list of fungal species, containing the entire phylogenetic classification for each genome included in the database. At the end of the process, an additional pipeline step allows the user to normalize the results by the length of the references included in the database. The obtained relative abundance profiles and the normalized number of hits for each sample are reported in tab-delimited files, along with histograms representing the fungal community, generated using the ‘base’ and ‘graphics’ R packages. The fungal reads, as identified above, are also provided in a .fastq file.

### Validation of the tool and comparison with other existing methods

A synthetic sample containing 1 million random sequences was generated using the EMBOSS makenucseq utility and analyzed to evaluate the HumanMycobiomeScan performance in avoiding the detection of false positives. Five additional mock communities composed of a set of 100-base reads were in silico generated. In particular, the latter contained a fungal fraction, consisting of 20 different species of varying abundance, 5 bacteria and the human genome, to simulate real metagenomes. The performance of HumanMycobiomeScan in correctly profiling the fungal community was compared with that of other available tools (i.e. the web-interfaces blastN [32] and MG-RAST [33]). All the genomes used to generate synthetic meta-communities are specified in Additional file 3. An evaluation dataset can be downloaded together with the tool at the project web page (https://sourceforge.net/projects/hmscan/).

### Case study: using HumanMycobiomeScan to profile the gut mycobiome of hunter-gatherers and Western subjects

Thirty-eight stool metagenomes from Rampelli et al. [34], including 11 metagenomes from Italian adults and 27 from the Hadza hunter-gatherers, were downloaded from the Sequence Read Archive [NCBI SRA; SRP056480, Bioproject ID PRJNA278393] and used to illustrate the performance and results of HumanMycobiomeScan. These metagenomes had been sequenced using the Illumina GAIIx platform, obtaining 0.9 Gbp of 2 × 100 bp paired-end reads. The entire metagenomic dataset was used to explore differences in the composition of fungal communities between groups of individuals relying on different subsistence strategies. No ethics committee approval was required to perform the analysis included in this study.

## Results

**Fig. 2:**
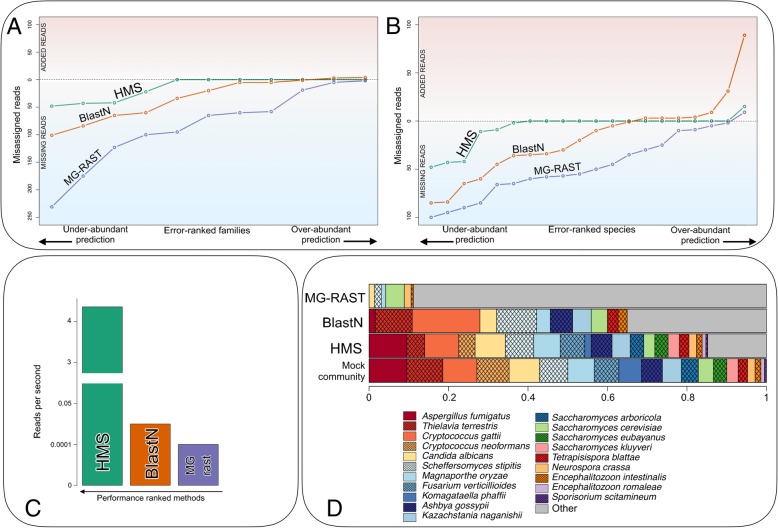
Comparison of HumanMycobiomeScan with other existing assignment methods. Five synthetic fungal communities were used to compare HumanMycobiomeScan (HMS) with BlastN [32] and MG-RAST [33]. The actual number of misassigned reads, including those under- or over-assigned, is reported at family (**a**) and species (**b**) level. The horizontal line in the plots represents the “expected” value, meaning that all reads for a specific taxon were assigned to the correct reference genome. Points below or above the line indicate a lower or higher number of reads assigned to a specific taxon compared to the expected value. **c** The number of reads processed per second working on a single CPU is shown. **d** A comparison between the actual relative abundances of a mock community taken as an example and those reconstructed using the various methods of analysis was carried out. The gray portion represents the fraction of misassigned reads

We first applied HumanMycobiomeScan to a synthetic sample containing random sequences to evaluate possible biases in the detection of false positives. As expected, no fungal hit was found but all sequences were filtered out in the first step of the procedure, when reads are screened against the database. We then evaluated the performance of the tool in investigating the fungal composition of five mock communities simulating a human-associated metagenome (i.e. including fungi, bacteria and the human genome). HumanMycobiomeScan correctly identified the 20 fungal species within the synthetic communities and estimated their abundance at different taxonomic levels (average number of misassigned reads: at family level, 8.5 (0.8% of reads); at species level, 14.1 (1.34% of reads)). All the species contained in the mock communities were detected and 86% of the fungal ones were assigned within 1.5% deviation from the expected value with the best overall prediction (Pearson r = 0.851, species-level Pearson *P* < 1 × 10^− 07^) (Fig. 2a–b). HumanMycobiomeScan was more accurate in profiling the mycobiome of synthetic metagenomes than other existing methods, with blastN showing the closest performance but being considerably slower (Fig. 2c). In particular, HumanMycobiomeScan performed the characterization at 4.36 reads per second on a standard single-processor, single-core system, which was several orders of magnitude faster than the other methods used for comparison. In addition, HumanMycobiomeScan showed a better prediction of fungal abundances (Fig. 2d). We then analyzed the results read by read, to understand how the approaches failed to assign the correct taxonomy. BlastN under- or over-estimated several fungal species, completely failed to detect 12 species (*Cryptococcus neoformans, Aspergillus fumigatus, Fusarium verticillioides, Komagataella phaffii, Saccharomyces arboricola, Candida albicans, Saccharomyces eubayanus, Magnaporthe oryzae, Saccharomyces kluyveri, Neurospora crassa, Encephalitozoon romaleae* and *Sporisorium scitamineum*), and assigned some reads to species that were not actually present in the mock community. The performance of MG-RAST was even more inaccurate, with nine reads out of 10 assigned to species not present in the mock samples. The greater accuracy of HumanMycobiomeScan and its computational speed in the assignment are probably due to the “two-step” process of the pipeline, which consists of two consecutive alignments of the reads to the reference database. The first alignment is performed at the very beginning, to identify candidate reads that are likely to belong to the fungal fraction of the ecosystem. The second alignment is subsequent to the filtering steps, as a validation and final assignment of the reads to the correct fungal taxonomy. Notably, this “two-step” approach, including filtering processes for bacterial and human reads, is the same as that used for the software ViromeScan [31] but designed, tested and optimized for mycobiome characterization. HumanMycobiomeScan was also able to assign the correct genus to reads for species not present in the databases, meaning that the tool is able to assign reads to the correct phylogeny when a related reference (i.e. belonging to the same genus) is present in the database.

**Fig. 3:**
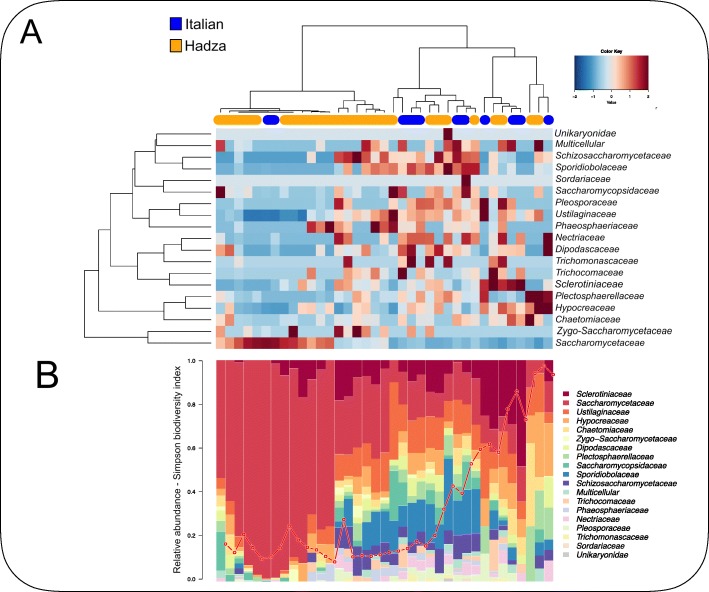
Characterization of the fungal fraction of the gut microbiome of populations with different subsistence strategies. **a** Family-level hierarchical Ward-linkage clustering based on the Spearman correlation coefficients of the fungal profiles of 37 metagenomes from Rampelli et al. [34], assigned using HumanMycobiomeScan. The study cohort includes 11 Italian subjects (in blue in the upper phylogenetic tree) and 26 Hadza hunter-gatherers from Tanzania (in orange). **b** The relative abundances of families are represented below the heatmap along with Simpson’s biodiversity index for each subject (red line)

In the second part of our analysis, we used HumanMycobiomeScan to explore the gut mycobiome of 38 subjects adhering to different subsistence strategies: 27 Hadza hunter-gatherers from Tanzania and 11 Western individuals from Italy. One Hadza subject (H4) was excluded from statistical analysis and graphical representations as no fungal hits were retrieved from shotgun sequences. HumanMycobiomeScan characterized the fungal community at different phylogenetic levels, detecting a total of 19 families and 65 species. Hierarchical clustering, performed using the Spearman distance and the Ward linkage on the family-level relative abundance profiles of the samples, revealed two distinct groups (*p* < 0.05, Fisher’s exact test) characterized by the dominance (relative abundance (rel. ab.) ≥ 30%) or not of the family *Saccharomycetaceae* (Fig. 3a-b). Interestingly, *Saccharomycetaceae* was almost the only fungal component detected in the feces of six subjects (rel. ab. > 90%). On the other hand, subjects with low abundance of *Saccharomycetaceae* (rel. ab. < 30%) showed greater biodiversity, with the concomitant presence of several fungal families, such as *Sclerotinaceae, Ustilaginaceae, Hypocreaceae, Dipodascaceae* and *Schizosaccharomycetaceae*. In spite of the profoundly different lifestyles of Hadza and Italians, in terms of both diet and contact with the environment [35], no significant differences in taxon relative abundance were found between the two populations. Future studies on larger worldwide cohorts, possibly including subjects practicing varying subsistence strategies and/or diseased patients, are needed to unravel the biological role of the human fungal microbiome in health and disease.

## Discussion

The HumanMycobiomeScan tool is specifically designed to detect fungal reads within complex human-associated microbiomes. In particular, it uses raw metagenomics reads in the .fastq format, generated by next-generation sequencing machines, and a read-mapping approach that allows high-speed profiling of the fungal community without any upstream process. The major advantage of such an approach is the preservation of all the information contained in the input files, otherwise lost using an assembly strategy [36]. This is especially relevant in the context of a metagenomic community, where fungal DNA is usually underrepresented due to the huge amount of bacterial and human sequences, making the assembly strategy really challenging. On the other hand, HumanMycobiomeScan, like other read-mapping approaches, is blind to fungal species whose genomes are not yet classified or are not closely related to those included in the database, which stresses the importance of updating databases when new genomes are released. In its current version, the tool is based on 66 fungal genomes out of the full 3.8 million estimated number of extant fungal species [37]. HumanMycobiomeScan is therefore not suitable for unexplored ecological niches but it is designed to profile well-characterized microbial communities (i.e. niches with known fungal genomes). HumanMycobiomeScan provides a detailed taxonomic description of the mycobiome under study, in terms of both raw number of hits and abundances. In particular, the raw read count output defines the richness and complexity of the fungal community within the source metagenome, whereas the abundance output describes the compositional structure in terms of relationships among fungal species. The HumanMycobiomeScan pipeline can be combined with other tools devoted to the characterization of metagenomic reads, such as ViromeScan (for viruses) [31] and MetaPhlAn (for bacteria) [38], thus allowing the user to get an overview of the microbiome (i.e. bacterial, viral and fungal counterparts) associated with a given environment.

## Conclusions

HumanMycobiomeScan opens up new possibilities in the metagenomics analysis of complex microbial ecosystems, extending in silico procedures to the characterization of the fungal component of microbiomes. By integrating the analysis with other tools already available to the scientific community, such as ViromeScan [31] and MetaPhlAn [37], the user can profile the viral, bacterial and fungal counterpart of a microbial community using the same shotgun sequencing data, with a considerable gain in cost and time. Furthermore, such an integrated approach allows retrieving a more complete picture of the analyzed microbiome, in terms of both microbial composition and richness of bacterial, viral and fungal sub-communities. A further advantage of HumanMycobiomeScan is the possibility of customizing the database by substituting or implementing the one supplied with the tool with fungal sequences of interest (see the instructions on the project web page). An update of the HumanMycobiomeScan database will be periodically performed to incorporate newly released fungal genomes.

### Availability and requirements

Project name: HumanMycobiomeScan

Project home page: https://sourceforge.net/projects/hmscan/

Operating system: command line on Linux or OS X

Programming language: Bash, R, Perl, Java

Other requirements: Bowtie2, BMTagger tools from NCBI, Picard tools. HMS can be run on a regular desktop computer, but a minimum of 16 GB of RAM is required. We strongly suggest that the tool is run on a cluster. To use the tool proficiently, a basic knowledge of command-line usage is recommended. Other information and options can be found in the help section of the tool.

Licence: FreBSD

Any restriction to use by non-academics: No

## Additional files

Additional file 1: NCBI ID and genome size for each genome included in the HumanMycobiomeScan database, Fungi_LITE. (PDF 160 kb)

Additional file 2: Genomes in the two databases are represented as pie charts color-coded by phylum assignment. (TIFF 158 kb)

Additional file 3: Genomes used to generate the synthetic meta-communities used in the HumanMycobiomeScan validation process. (PDF 22 kb)

## Data Availability

The dataset analyzed in the present study is available at the Sequence Read Archive [NCBI SRA; SRP056480, Bioproject ID PRJNA278393].

## Abbreviations

BMTagger: Human best match tagger
HMP: Human microbiome project
