# Supplementary material for: HumanMycobiomeScan: a new bioinformatics tool for the characterization of the fungal fraction in metagenomic samples
Source: BMC Genomics. 2019 Jun 15;20:496. doi: 10.1186/s12864-019-5883-y (PMC6570844; doi:10.1186/s12864-019-5883-y)
Supplement: Supplementary file 1 — NCBI ID and genome size for each genome included in the HumanMycobiomeScan database, Fungi_LITE. (PDF 160 kb) [file 12864_2019_5883_MOESM1_ESM.pdf]

CR382121.1 Kluyveromyces lactis strain NRRL Y-1140 chromosome A complete sequence
1062590
CR382122.1 Kluyveromyces lactis strain NRRL Y-1140 chromosome B complete sequence
1320834
CR382123.1 Kluyveromyces lactis strain NRRL Y-1140 chromosome C complete sequence
1753957
CR382124.1 Kluyveromyces lactis strain NRRL Y-1140 chromosome D complete sequence
1715506
CR382125.1 Kluyveromyces lactis strain NRRL Y-1140 chromosome E complete sequence
2234072
CR382126.1 Kluyveromyces lactis strain NRRL Y-1140 chromosome F complete sequence
2602197
CR382127.1 Yarrowia lipolytica CLIB122 chromosome A complete sequence
2303261
CR382128.1 Yarrowia lipolytica CLIB122 chromosome B complete sequence
3066374
CR382129.1 Yarrowia lipolytica CLIB122 chromosome C complete sequence
3272609
CR382130.1 Yarrowia lipolytica CLIB122 chromosome D complete sequence
3633272
CR382131.1 Yarrowia lipolytica CLIB122 chromosome E complete sequence
4224103
CR382132.1 Yarrowia lipolytica CLIB122 chromosome F complete sequence
4003362
CR380947.2 Candida glabrata strain CBS138 chromosome A complete sequence
491328
CR380948.1 Candida glabrata strain CBS138 chromosome B complete sequence
502101
CR380949.1 Candida glabrata strain CBS138 chromosome C complete sequence
558804
CR380950.1 Candida glabrata strain CBS138 chromosome D complete sequence
651701
CR380951.2 Candida glabrata strain CBS138 chromosome E complete sequence
687738
CR380952.1 Candida glabrata strain CBS138 chromosome F complete sequence
927101
CR380953.1 Candida glabrata strain CBS138 chromosome G complete sequence
992211
CR380954.1 Candida glabrata strain CBS138 chromosome H complete sequence
1050361
CR380955.2 Candida glabrata strain CBS138 chromosome I complete sequence
1100349
CR380956.2 Candida glabrata strain CBS138 chromosome J complete sequence
1195132
CR380957.2 Candida glabrata strain CBS138 chromosome K complete sequence
1302831
CR380958.2 Candida glabrata strain CBS138 chromosome L complete sequence
1455689
CR380959.2 Candida glabrata strain CBS138 chromosome M complete sequence
1402899
CU329670.1 Schizosaccharomyces pombe chromosome I, complete sequence
5579133
CU329671.1 Schizosaccharomyces pombe chromosome II, complete sequence
4539804
CU329672.1 Schizosaccharomyces pombe chromosome III, complete sequence
2452883
X54421.1 Schizosaccharomyces pombe complete mitochondrial genome
19431
CR382133.2 Debaryomyces hansenii CBS767 chromosome A complete sequence
1249940

CR382134.2 Debaryomyces hansenii CBS767 chromosome B complete sequence
1344482
CR382135.2 Debaryomyces hansenii CBS767 chromosome C complete sequence
1587442
CR382136.2 Debaryomyces hansenii CBS767 chromosome A complete sequence
1606296
CR382137.2 Debaryomyces hansenii CBS767 chromosome E complete sequence
2007515
CR382138.2 Debaryomyces hansenii CBS767 chromosome F complete sequence
2305761
CR382139.2 Debaryomyces hansenii CBS767 chromosome G complete sequence
2051050
CU928173.1 Zygosaccharomyces rouxii strain CBS732 chromosome A complete sequence
1114666
CU928174.1 Zygosaccharomyces rouxii strain CBS732 chromosome B complete sequence
1388208
CU928175.1 Zygosaccharomyces rouxii strain CBS732 chromosome C complete sequence
1464093
CU928176.1 Zygosaccharomyces rouxii strain CBS732 chromosome D complete sequence
1496342
CU928181.1 Zygosaccharomyces rouxii strain CBS732 chromosome E complete sequence
881646
CU928178.1 Zygosaccharomyces rouxii strain CBS732 chromosome F complete sequence
1554288
CU928179.1 Zygosaccharomyces rouxii strain CBS732 chromosome G complete sequence
1865392
FM992695.1 Candida dubliniensis CD36 chromosome R, complete sequence
2267510
FM992688.1 Candida dubliniensis CD36 chromosome 1, complete sequence
3214061
FM992689.1 Candida dubliniensis CD36 chromosome 2, complete sequence
2289089
FM992690.1 Candida dubliniensis CD36 chromosome 3, complete sequence
1863824
FM992691.1 Candida dubliniensis CD36 chromosome 4, complete sequence
1641709
FM992692.1 Candida dubliniensis CD36 chromosome 5, complete sequence
1245899
FM992693.1 Candida dubliniensis CD36 chromosome 6, complete sequence
1073895
FM992694.1 Candida dubliniensis CD36 chromosome 7, complete sequence
1022435
FN392319.1 Pichia pastoris GS115 chromosome 1, complete sequence
2798491
FN392320.1 Pichia pastoris GS115 chromosome 2, complete sequence
2394163
FN392321.1 Pichia pastoris GS115 chromosome 3, complete sequence
2245428
FN392322.1 Pichia pastoris GS115 chromosome 4, complete sequence
1778296
AE016814.2 Ashbya gossypii ATCC 10895 chromosome I, complete sequence
693414
AE016815.5 Ashbya gossypii ATCC 10895 chromosome II, complete sequence
870771
AE016816.3 Ashbya gossypii ATCC 10895 chromosome III, complete sequence
907494
AE016817.6 Ashbya gossypii ATCC 10895 chromosome IV, complete sequence
1467287
AE016818.2 Ashbya gossypii ATCC 10895 chromosome V, complete sequence
1519140

AE016819.5 Ashbya gossypii ATCC 10895 chromosome VI, complete sequence
1836693
AE016820.4 Ashbya gossypii ATCC 10895 chromosome VII, complete sequence
1800949
AE016821.1 Ashbya gossypii (= Eremothecium gossypii) ATCC 10895 mitochondrion, complete genome
23564
AE017341.1 Cryptococcus neoformans var. neoformans JEC21 chromosome 1, complete sequence
2300533
X59720.2 S.cerevisiae chromosome III complete DNA sequence
316613
D50617.1 Saccharomyces cerevisiae DNA, chromosome VI, complete genome
270148
CU928165.1 Lachancea thermotolerans CBS 6340 chromosome A complete sequence
687718
CU928166.1 Lachancea thermotolerans CBS 6340 chromosome B complete sequence
893706
CU928167.1 Lachancea thermotolerans CBS 6340 chromosome C complete sequence
999246
CU928168.1 Lachancea thermotolerans CBS 6340 chromosome D complete sequence
1513537
CU928169.1 Lachancea thermotolerans CBS 6340 chromosome E complete sequence
1521774
CU928170.1 Lachancea thermotolerans CBS 6340 chromosome F complete sequence
1632914
CU928171.1 Lachancea thermotolerans CBS 6340 chromosome G complete sequence
1720065
CU928180.1 Lachancea thermotolerans CBS 6340 chromosome H complete sequence
1423902
CP009805.1 Botrytis cinerea B05.10 chromosome BCIN01, complete sequence
4109373
CP009806.1 Botrytis cinerea B05.10 chromosome BCIN02, complete sequence
3341473
CP009807.1 Botrytis cinerea B05.10 chromosome BCIN03, complete sequence
3226611
CP009808.1 Botrytis cinerea B05.10 chromosome BCIN04, complete sequence
2468882
CP009809.1 Botrytis cinerea B05.10 chromosome BCIN05, complete sequence
2959378
CP009810.1 Botrytis cinerea B05.10 chromosome BCIN06, complete sequence
2725906
CP009811.1 Botrytis cinerea B05.10 chromosome BCIN07, complete sequence
2652353
CP009812.1 Botrytis cinerea B05.10 chromosome BCIN08, complete sequence
2617329
CP009813.1 Botrytis cinerea B05.10 chromosome BCIN09, complete sequence
2547566
CP009814.1 Botrytis cinerea B05.10 chromosome BCIN10, complete sequence
2419276
CP009815.1 Botrytis cinerea B05.10 chromosome BCIN11, complete sequence
2359939
CP009816.1 Botrytis cinerea B05.10 chromosome BCIN12, complete sequence
2352958
CP009817.1 Botrytis cinerea B05.10 chromosome BCIN13, complete sequence
2257609
CP009818.1 Botrytis cinerea B05.10 chromosome BCIN14, complete sequence
2138025
CP009819.1 Botrytis cinerea B05.10 chromosome BCIN15, complete sequence
2027721

CP009820.1 Botrytis cinerea B05.10 chromosome BCIN16, complete sequence
1969743
CP009821.1 Botrytis cinerea B05.10 chromosome BCIN17, complete sequence
247158
CP009822.1 Botrytis cinerea B05.10 chromosome BCIN18, complete sequence
208766
BK006935.2 TPA_inf: Saccharomyces cerevisiae S288C chromosome I, complete sequence
230218
BK006936.2 TPA_inf: Saccharomyces cerevisiae S288C chromosome II, complete sequence
813184
BK006937.2 TPA_inf: Saccharomyces cerevisiae S288C chromosome III, complete sequence
316620
BK006938.2 TPA_inf: Saccharomyces cerevisiae S288C chromosome IV, complete sequence
1531933
BK006939.2 TPA_inf: Saccharomyces cerevisiae S288C chromosome V, complete sequence
576874
BK006940.2 TPA_inf: Saccharomyces cerevisiae S288C chromosome VI, complete sequence
270161
BK006941.2 TPA_inf: Saccharomyces cerevisiae S288C chromosome VII, complete sequence
1090940
BK006934.2 TPA_inf: Saccharomyces cerevisiae S288C chromosome VIII, complete sequence
562643
BK006942.2 TPA_inf: Saccharomyces cerevisiae S288C chromosome IX, complete sequence
439888
BK006943.2 TPA_inf: Saccharomyces cerevisiae S288C chromosome X, complete sequence
745751
BK006944.2 TPA_inf: Saccharomyces cerevisiae S288C chromosome XI, complete sequence
666816
BK006945.2 TPA_inf: Saccharomyces cerevisiae S288C chromosome XII, complete sequence
1078177
BK006946.2 TPA_inf: Saccharomyces cerevisiae S288C chromosome XIII, complete sequence
924431
BK006947.3 TPA_inf: Saccharomyces cerevisiae S288C chromosome XIV, complete sequence
784333
BK006948.2 TPA_inf: Saccharomyces cerevisiae S288C chromosome XV, complete sequence
1091291
BK006949.2 TPA_inf: Saccharomyces cerevisiae S288C chromosome XVI, complete sequence
948066
CP001942.1 Encephalitozoon intestinalis ATCC 50506 chromosome I, complete sequence
160332
CP001943.1 Encephalitozoon intestinalis ATCC 50506 chromosome II, complete sequence
175776
CP001944.1 Encephalitozoon intestinalis ATCC 50506 chromosome III, complete sequence
176815
CP001945.1 Encephalitozoon intestinalis ATCC 50506 chromosome IV, complete sequence
193740
CP001946.1 Encephalitozoon intestinalis ATCC 50506 chromosome V, complete sequence
196642
CP001947.1 Encephalitozoon intestinalis ATCC 50506 chromosome VI, complete sequence
198217
CP001948.1 Encephalitozoon intestinalis ATCC 50506 chromosome VII, complete sequence
205935
CP001949.1 Encephalitozoon intestinalis ATCC 50506 chromosome VIII, complete sequence
204910
CP001951.1 Encephalitozoon intestinalis ATCC 50506 chromosome X, complete sequence
234890
CP001952.1 Encephalitozoon intestinalis ATCC 50506 chromosome XI, complete sequence
236244
CP003820.1 Cryptococcus neoformans var. grubii H99 chromosome 1, complete sequence
2291499

CP003821.1 Cryptococcus neoformans var. grubii H99 chromosome 2, complete sequence
1621675
CP003822.1 Cryptococcus neoformans var. grubii H99 chromosome 3, complete sequence
1575141
CP003823.1 Cryptococcus neoformans var. grubii H99 chromosome 4, complete sequence
1084805
CP003824.1 Cryptococcus neoformans var. grubii H99 chromosome 5, complete sequence
1814975
CP003825.1 Cryptococcus neoformans var. grubii H99 chromosome 6, complete sequence
1422463
CP003826.1 Cryptococcus neoformans var. grubii H99 chromosome 7, complete sequence
1399503
CP003827.1 Cryptococcus neoformans var. grubii H99 chromosome 8, complete sequence
1398693
CP003828.1 Cryptococcus neoformans var. grubii H99 chromosome 9, complete sequence
1186808
CP003829.1 Cryptococcus neoformans var. grubii H99 chromosome 10, complete sequence
1059964
CP003830.1 Cryptococcus neoformans var. grubii H99 chromosome 11, complete sequence
1561994
CP003831.1 Cryptococcus neoformans var. grubii H99 chromosome 12, complete sequence
774062
CP003832.1 Cryptococcus neoformans var. grubii H99 chromosome 13, complete sequence
756744
CP003833.2 Cryptococcus neoformans var. grubii H99 chromosome 14, complete sequence
942867
CP003834.1 Cryptococcus neoformans var. grubii H99 mitochondrion, complete genome
24919
KC683708.1 Neurospora crassa OR74A mitochondrion, complete genome
64840
AP007176.1 Aspergillus oryzae RIB40 mitochondrial DNA, complete genome, AOmito_02s
29202
CP000286.1 Cryptococcus gattii WM276 chromosome A, complete sequence
1984823
CP000287.1 Cryptococcus gattii WM276 chromosome B, complete sequence
2187695
CP000288.1 Cryptococcus gattii WM276 chromosome C, complete sequence
1961512
CP000289.1 Cryptococcus gattii WM276 chromosome D, complete sequence
2233618
CP000290.1 Cryptococcus gattii WM276 chromosome E, complete sequence
1333124
CP000291.1 Cryptococcus gattii WM276 chromosome F, complete sequence
1325755
CP000292.1 Cryptococcus gattii WM276 chromosome G, complete sequence
1324677
CP000293.1 Cryptococcus gattii WM276 chromosome H, complete sequence
1265488
CP000294.1 Cryptococcus gattii WM276 chromosome I, complete sequence
989306
CP000295.1 Cryptococcus gattii WM276 chromosome J, complete sequence
522727
CP000296.1 Cryptococcus gattii WM276 chromosome K, complete sequence
1040760
CP000297.1 Cryptococcus gattii WM276 chromosome L, complete sequence
820445
CP000298.1 Cryptococcus gattii WM276 chromosome M, complete sequence
705823
CP000299.1 Cryptococcus gattii WM276 chromosome N, complete sequence
679007

CP000496.1 Scheffersomyces stipitis CBS 6054 chromosome 2, complete sequence
2740984
CP000497.1 Scheffersomyces stipitis CBS 6054 chromosome 3, complete sequence
1841851
CP000498.1 Scheffersomyces stipitis CBS 6054 chromosome 4, complete sequence
1803401
CP000499.1 Scheffersomyces stipitis CBS 6054 chromosome 5, complete sequence
1725948
CP000500.1 Scheffersomyces stipitis CBS 6054 chromosome 6, complete sequence
1724953
CP000501.1 Scheffersomyces stipitis CBS 6054 chromosome 7, complete sequence
1114415
CP000502.1 Scheffersomyces stipitis CBS 6054 chromosome 8, complete sequence
979380
FR839628.1 Pichia pastoris CBS 7435 chromosome 1, complete replicon sequence
2891190
FR839629.1 Pichia pastoris CBS 7435 chromosome 2, complete replicon sequence
2399323
FR839630.1 Pichia pastoris CBS 7435 chromosome 3, complete replicon sequence
2256069
FR839631.1 Pichia pastoris CBS 7435 chromosome 4, complete replicon sequence
1820458
FR839632.1 Pichia pastoris CBS 7435 mitochondrion, complete replicon sequence
35683
CP003002.1 Myceliophthora thermophila ATCC 42464 chromosome 1, complete sequence
10931058
CP003003.1 Myceliophthora thermophila ATCC 42464 chromosome 2, complete sequence
5454242
CP003004.1 Myceliophthora thermophila ATCC 42464 chromosome 3, complete sequence
5062665
CP003005.1 Myceliophthora thermophila ATCC 42464 chromosome 4, complete sequence
4710208
CP003006.1 Myceliophthora thermophila ATCC 42464 chromosome 5, complete sequence
4342007
CP003007.1 Myceliophthora thermophila ATCC 42464 chromosome 6, complete sequence
4127677
CP003008.1 Myceliophthora thermophila ATCC 42464 chromosome 7, complete sequence
4116359
CP003009.1 Thielavia terrestris NRRL 8126 chromosome 1, complete sequence
10101509
CP003010.1 Thielavia terrestris NRRL 8126 chromosome 2, complete sequence
9477512
CP003011.1 Thielavia terrestris NRRL 8126 chromosome 3, complete sequence
4786945
CP003012.1 Thielavia terrestris NRRL 8126 chromosome 4, complete sequence
4578922
CP003013.1 Thielavia terrestris NRRL 8126 chromosome 5, complete sequence
4396881
CP003014.1 Thielavia terrestris NRRL 8126 chromosome 6, complete sequence
3570487
HE580267.1 Naumovozyma dairenensis CBS 421 chromosome 1, complete genome
2054635
HE580268.1 Naumovozyma dairenensis CBS 421 chromosome 2, complete genome
1549475
HE580269.1 Naumovozyma dairenensis CBS 421 chromosome 3, complete genome
1536206
HE580270.1 Naumovozyma dairenensis CBS 421 chromosome 4, complete genome
1230053
HE580271.1 Naumovozyma dairenensis CBS 421 chromosome 5, complete genome
1184459

HE580272.1 Naumovozyma dairenensis CBS 421 chromosome 6, complete genome
1138463
HE580273.2 Naumovozyma dairenensis CBS 421 chromosome 7, complete genome
1565278
HE580274.1 Naumovozyma dairenensis CBS 421 chromosome 8, complete genome
1004037
HE580275.1 Naumovozyma dairenensis CBS 421 chromosome 9, complete genome
786484
HE580276.1 Naumovozyma dairenensis CBS 421 chromosome 10, complete genome
782356
HE580277.1 Naumovozyma dairenensis CBS 421 chromosome 11, complete genome
696134
FQ311430.1 Sporisorium reilianum SRZ2 chromosome 1 complete DNA sequence
2448206
FQ311441.1 Sporisorium reilianum SRZ2 chromosome 2 complete DNA sequence
1791267
FQ311452.1 Sporisorium reilianum SRZ2 chromosome 3 complete DNA sequence
1534569
FQ311463.1 Sporisorium reilianum SRZ2 chromosome 4 complete DNA sequence
772363
FQ311470.1 Sporisorium reilianum SRZ2 chromosome 5 complete DNA sequence
730625
FQ311471.1 Sporisorium reilianum SRZ2 chromosome 6 complete DNA sequence
976264
FQ311472.1 Sporisorium reilianum SRZ2 chromosome 7 complete DNA sequence
880029
FQ311473.1 Sporisorium reilianum SRZ2 chromosome 8 complete DNA sequence
760034
FQ311474.1 Sporisorium reilianum SRZ2 chromosome 9 complete DNA sequence
670125
FQ311431.1 Sporisorium reilianum SRZ2 chromosome 10 complete DNA sequence
642761
FQ311432.1 Sporisorium reilianum SRZ2 chromosome 11 complete DNA sequence
636629
FQ311433.1 Sporisorium reilianum SRZ2 chromosome 12 complete DNA sequence
565009
FQ311434.1 Sporisorium reilianum SRZ2 chromosome 13 complete DNA sequence
555666
FQ311435.1 Sporisorium reilianum SRZ2 chromosome 14 complete DNA sequence
562274
FQ311436.1 Sporisorium reilianum SRZ2 chromosome 15 complete DNA sequence
562189
FQ311437.1 Sporisorium reilianum SRZ2 chromosome 16 complete DNA sequence
478111
FQ311438.1 Sporisorium reilianum SRZ2 chromosome 17 complete DNA sequence
534508
FQ311439.1 Sporisorium reilianum SRZ2 chromosome 18 complete DNA sequence
518890
FQ311440.1 Sporisorium reilianum SRZ2 chromosome 19 complete DNA sequence
521053
FQ311442.1 Sporisorium reilianum SRZ2 chromosome 20 complete DNA sequence
1115737
FQ311443.1 Sporisorium reilianum SRZ2 chromosome 21 complete DNA sequence
429375
FQ311444.1 Sporisorium reilianum SRZ2 chromosome 22 complete DNA sequence
385084
FQ311445.1 Sporisorium reilianum SRZ2 chromosome 23 complete DNA sequence
263978
FQ311446.1 Sporisorium reilianum SRZ2 contig_5.00010 complete DNA sequence
1151

FQ311447.1 Sporisorium reilianum SRZ2 contig_5.00013 complete DNA sequence
1431
FQ311448.1 Sporisorium reilianum SRZ2 contig_5.00023 complete DNA sequence
1758
FQ311449.1 Sporisorium reilianum SRZ2 contig_5.00029 complete DNA sequence
1581
FQ311450.1 Sporisorium reilianum SRZ2 contig_5.00032 complete DNA sequence
1796
FQ311451.1 Sporisorium reilianum SRZ2 contig_5.00045 complete DNA sequence
1309
FQ311453.1 Sporisorium reilianum SRZ2 contig_5.00046 complete DNA sequence
721
FQ311454.1 Sporisorium reilianum SRZ2 contig_5.00050 complete DNA sequence
2378
FQ311455.1 Sporisorium reilianum SRZ2 contig_5.00055 complete DNA sequence
4673
FQ311456.1 Sporisorium reilianum SRZ2 contig_5.00068 complete DNA sequence
1537
FQ311457.1 Sporisorium reilianum SRZ2 contig_5.00069 complete DNA sequence
864
FQ311458.1 Sporisorium reilianum SRZ2 contig_5.00075 complete DNA sequence
19032
FQ311459.1 Sporisorium reilianum SRZ2 contig_5.00080 complete DNA sequence
1970
FQ311460.1 Sporisorium reilianum SRZ2 contig_5.00081 complete DNA sequence
1618
FQ311461.1 Sporisorium reilianum SRZ2 contig_5.00088 complete DNA sequence
1446
FQ311462.1 Sporisorium reilianum SRZ2 sr_contig_5.00092 complete DNA sequence
759
FQ311464.1 Sporisorium reilianum SRZ2 sr_contig_5.00094 complete DNA sequence
796
FQ311465.1 Sporisorium reilianum SRZ2 contig_5.00095 complete DNA sequence
776
FQ311466.1 Sporisorium reilianum SRZ2 contig_5.00096 complete DNA sequence
3131
FQ311467.1 Sporisorium reilianum SRZ2 contig_5.00104 complete DNA sequence
1823
FQ311468.1 Sporisorium reilianum SRZ2 contig_5.00115 complete DNA sequence
1082
CP002497.1 Eremothecium cymbalariae DBVPG#7215 chromosome 1, complete sequence
1110245
CP002498.1 Eremothecium cymbalariae DBVPG#7215 chromosome 2, complete sequence
1601921
CP002499.1 Eremothecium cymbalariae DBVPG#7215 chromosome 3, complete sequence
1193613
CP002500.1 Eremothecium cymbalariae DBVPG#7215 chromosome 4, complete sequence
1540972
CP002501.1 Eremothecium cymbalariae DBVPG#7215 chromosome 5, complete sequence
1385851
CP002502.1 Eremothecium cymbalariae DBVPG#7215 chromosome 6, complete sequence
959278
CP002503.1 Eremothecium cymbalariae DBVPG#7215 chromosome 7, complete sequence
980088
CP002504.1 Eremothecium cymbalariae DBVPG#7215 chromosome 8, complete sequence
897456
HE612856.1 Tetrapisispora phaffii CBS 4417 chromosome 1, complete genome
1377933
HE612857.1 Tetrapisispora phaffii CBS 4417 chromosome 2, complete genome
1164785

HE612858.1 Tetrapisispora phaffii CBS 4417 chromosome 3, complete genome
1096211
HE612859.1 Tetrapisispora phaffii CBS 4417 chromosome 4, complete genome
1042069
HE612860.1 Tetrapisispora phaffii CBS 4417 chromosome 5, complete genome
854350
HE612861.1 Tetrapisispora phaffii CBS 4417 chromosome 6, complete genome
815984
HE612862.1 Tetrapisispora phaffii CBS 4417 chromosome 7, complete genome
808783
HE612863.1 Tetrapisispora phaffii CBS 4417 chromosome 8, complete genome
752451
HE612864.1 Tetrapisispora phaffii CBS 4417 chromosome 9, complete genome
740259
HE612865.1 Tetrapisispora phaffii CBS 4417 chromosome 10, complete genome
713392
HE612866.1 Tetrapisispora phaffii CBS 4417 chromosome 11, complete genome
536385
HE612867.1 Tetrapisispora phaffii CBS 4417 chromosome 12, complete genome
504740
HE612868.1 Tetrapisispora phaffii CBS 4417 chromosome 13, complete genome
452480
HE612869.1 Tetrapisispora phaffii CBS 4417 chromosome 14, complete genome
432466
HE612870.1 Tetrapisispora phaffii CBS 4417 chromosome 15, complete genome
413379
HE612871.1 Tetrapisispora phaffii CBS 4417 chromosome 16, complete genome
394523
HE576752.1 Naumovozyma castellii CBS 4309 chromosome 1, complete genome
3019861
HE576753.1 Naumovozyma castellii CBS 4309 chromosome 2, complete genome
1756361
HE576754.1 Naumovozyma castellii CBS 4309 chromosome 3, complete genome
1245273
HE576755.1 Naumovozyma castellii CBS 4309 chromosome 4, complete genome
958728
HE576756.1 Naumovozyma castellii CBS 4309 chromosome 5, complete genome
835708
HE576757.1 Naumovozyma castellii CBS 4309 chromosome 6, complete genome
828437
HE576758.1 Naumovozyma castellii CBS 4309 chromosome 7, complete genome
804126
HE576759.1 Naumovozyma castellii CBS 4309 chromosome 8, complete genome
699326
HE576760.1 Naumovozyma castellii CBS 4309 chromosome 9, complete genome
603245
HE576761.1 Naumovozyma castellii CBS 4309 chromosome 10, complete genome
468474
HE616742.1 Torulaspora delbrueckii CBS 1146 chromosome 1, complete genome
1418021
HE616743.1 Torulaspora delbrueckii CBS 1146 chromosome 2, complete genome
1354397
HE616744.1 Torulaspora delbrueckii CBS 1146 chromosome 3, complete genome
1292049
HE616745.1 Torulaspora delbrueckii CBS 1146 chromosome 4, complete genome
1218070
HE616746.1 Torulaspora delbrueckii CBS 1146 chromosome 5, complete genome
1086982
HE616747.1 Torulaspora delbrueckii CBS 1146 chromosome 6, complete genome
1070623

HE616748.1 Torulaspora delbrueckii CBS 1146 chromosome 7, complete genome
946563
HE616749.1 Torulaspora delbrueckii CBS 1146 chromosome 8, complete genome
833973
CP002713.1 Encephalitozoon hellem ATCC 50504 chromosome I, complete sequence
161584
CP002714.1 Encephalitozoon hellem ATCC 50504 chromosome II, complete sequence
179949
CP002715.1 Encephalitozoon hellem ATCC 50504 chromosome III, complete sequence
182562
CP002716.1 Encephalitozoon hellem ATCC 50504 chromosome IV, complete sequence
196397
CP002717.1 Encephalitozoon hellem ATCC 50504 chromosome V, complete sequence
201478
CP002718.1 Encephalitozoon hellem ATCC 50504 chromosome VI, complete sequence
200132
CP002719.1 Encephalitozoon hellem ATCC 50504 chromosome VII, complete sequence
213233
CP002720.1 Encephalitozoon hellem ATCC 50504 chromosome VIII, complete sequence
203699
CP002721.1 Encephalitozoon hellem ATCC 50504 chromosome IXa, complete sequence
167643
CP002722.1 Encephalitozoon hellem ATCC 50504 chromosome IXb, complete sequence
66626
CP002723.1 Encephalitozoon hellem ATCC 50504 chromosome X, complete sequence
240865
CP002724.1 Encephalitozoon hellem ATCC 50504 chromosome XI, complete sequence
237616
CP003518.1 Encephalitozoon romaleae SJ-2008 chromosome I, complete sequence
146158
CP003519.1 Encephalitozoon romaleae SJ-2008 chromosome II, complete sequence
173763
CP003520.1 Encephalitozoon romaleae SJ-2008 chromosome III, complete sequence
179400
CP003521.1 Encephalitozoon romaleae SJ-2008 chromosome IV, complete sequence
197308
CP003522.1 Encephalitozoon romaleae SJ-2008 chromosome V, complete sequence
191809
CP003523.1 Encephalitozoon romaleae SJ-2008 chromosome VI, complete sequence
194799
CP003524.1 Encephalitozoon romaleae SJ-2008 chromosome VII, complete sequence
204533
CP003525.1 Encephalitozoon romaleae SJ-2008 chromosome VIII, complete sequence
207682
CP003529.1 Encephalitozoon romaleae SJ-2008 chromosome X, complete sequence
230122
CP003530.1 Encephalitozoon romaleae SJ-2008 chromosome XI, complete sequence
231554
CP015461.1 Agaricus bisporus var. bisporus H97 chromosome 5, complete sequence
2550681
CP015462.1 Agaricus bisporus var. bisporus H97 chromosome 6, complete sequence
2329815
HE650821.1 Kazachstania africana CBS 2517 chromosome 1, complete genome
1757399
HE650822.1 Kazachstania africana CBS 2517 chromosome 2, complete genome
1485491
HE650823.1 Kazachstania africana CBS 2517 chromosome 3, complete genome
1340033
HE650824.1 Kazachstania africana CBS 2517 chromosome 4, complete genome
1026673

HE650825.1 Kazachstania africana CBS 2517 chromosome 5, complete genome
925516
HE650826.1 Kazachstania africana CBS 2517 chromosome 6, complete genome
888590
HE650827.1 Kazachstania africana CBS 2517 chromosome 7, complete genome
812636
HE650828.1 Kazachstania africana CBS 2517 chromosome 8, complete genome
738805
HE650829.1 Kazachstania africana CBS 2517 chromosome 9, complete genome
607727
HE650830.1 Kazachstania africana CBS 2517 chromosome 10, complete genome
578118
HE650831.1 Kazachstania africana CBS 2517 chromosome 11, complete genome
547686
HE650832.1 Kazachstania africana CBS 2517 chromosome 12, complete genome
421466
FO082059.1 Millerozyma farinosa CBS 7064 chromosome A complete sequence
1055225
FO082058.1 Millerozyma farinosa CBS 7064 chromosome B complete sequence
1063002
FO082057.1 Pichia sorbitophila strain CBS 7064 chromosome C complete sequence
1095816
FO082056.1 Pichia sorbitophila strain CBS 7064 chromosome D complete sequence
1102865
FO082055.1 Pichia sorbitophila strain CBS 7064 chromosome E complete sequence
1307235
FO082054.1 Pichia sorbitophila strain CBS 7064 chromosome F complete sequence
1407903
FO082053.1 Pichia sorbitophila strain CBS 7064 chromosome G complete sequence
1423303
FO082052.1 Pichia sorbitophila strain CBS 7064 chromosome H complete sequence
1423303
FO082051.1 Pichia sorbitophila strain CBS 7064 chromosome I complete sequence
1666063
FO082050.1 Pichia sorbitophila strain CBS 7064 chromosome J complete sequence
1803284
FO082049.1 Pichia sorbitophila strain CBS 7064 chromosome K complete sequence
1933849
FO082048.1 Pichia sorbitophila strain CBS 7064 chromosome L complete sequence
1933849
FO082047.1 Pichia sorbitophila strain CBS 7064 chromosome M complete sequence
2121241
FO082046.1 Pichia sorbitophila strain CBS 7064 chromosome N complete sequence
2122704
HE806316.1 Tetrapisispora blattae CBS 6284 chromosome 1, complete genome
2697332
HE806317.1 Tetrapisispora blattae CBS 6284 chromosome 2, complete genome
2450908
HE806318.1 Tetrapisispora blattae CBS 6284 chromosome 3, complete genome
1779678
HE806319.1 Tetrapisispora blattae CBS 6284 chromosome 4, complete genome
1449145
HE806320.1 Tetrapisispora blattae CBS 6284 chromosome 5, complete genome
1324051
HE806321.1 Tetrapisispora blattae CBS 6284 chromosome 6, complete genome
1053862
HE806322.1 Tetrapisispora blattae CBS 6284 chromosome 7, complete genome
986391
HE806323.1 Tetrapisispora blattae CBS 6284 chromosome 8, complete genome
956377

HE806324.1 Tetrapisispora blattae CBS 6284 chromosome 9, complete genome
857919
HE806325.1 Tetrapisispora blattae CBS 6284 chromosome 10, complete genome
492930
HE978314.1 Kazachstania naganishii CBS 8797 chromosome 1, complete genome
1290777
HE978315.1 Kazachstania naganishii CBS 8797 chromosome 2, complete genome
1410756
HE978316.1 Kazachstania naganishii CBS 8797 chromosome 3, complete genome
1310278
HE978317.1 Kazachstania naganishii CBS 8797 chromosome 4, complete genome
993246
HE978318.1 Kazachstania naganishii CBS 8797 chromosome 5, complete genome
856010
HE978319.1 Kazachstania naganishii CBS 8797 chromosome 6, complete genome
770551
HE978320.1 Kazachstania naganishii CBS 8797 chromosome 7, complete genome
780220
HE978321.1 Kazachstania naganishii CBS 8797 chromosome 8, complete genome
724298
HE978322.1 Kazachstania naganishii CBS 8797 chromosome 9, complete genome
628580
HE978323.1 Kazachstania naganishii CBS 8797 chromosome 10, complete genome
586734
HE978324.1 Kazachstania naganishii CBS 8797 chromosome 11, complete genome
538562
HE978325.1 Kazachstania naganishii CBS 8797 chromosome 12, complete genome
450952
HE978326.1 Kazachstania naganishii CBS 8797 chromosome 13, complete genome
504857
CP009075.1 Verticillium dahliae JR2 chromosome 1, complete sequence
9275483
CP009079.1 Verticillium dahliae JR2 chromosome 2, complete sequence
4277765
CP009077.1 Verticillium dahliae JR2 chromosome 3, complete sequence
4168633
CP009080.1 Verticillium dahliae JR2 chromosome 4, complete sequence
4086908
CP009078.1 Verticillium dahliae JR2 chromosome 5, complete sequence
4171808
CP009076.1 Verticillium dahliae JR2 chromosome 6, complete sequence
3530890
CP009082.1 Verticillium dahliae JR2 chromosome 7, complete sequence
3277570
CP009081.1 Verticillium dahliae JR2 chromosome 8, complete sequence
3361230
CP006020.1 Saccharomycetaceae sp. 'Ashbya aceri' chromosome I, complete sequence
809179
CP006021.1 Saccharomycetaceae sp. 'Ashbya aceri' chromosome II, complete sequence
1019532
CP006022.1 Saccharomycetaceae sp. 'Ashbya aceri' chromosome III, complete sequence
1493473
CP006023.1 Saccharomycetaceae sp. 'Ashbya aceri' chromosome IV, complete sequence
1099572
CP006024.1 Saccharomycetaceae sp. 'Ashbya aceri' chromosome V, complete sequence
1513917
CP006025.1 Saccharomycetaceae sp. 'Ashbya aceri' chromosome VI, complete sequence
1334226
CP006026.1 Saccharomycetaceae sp. 'Ashbya aceri' chromosome VII, complete sequence
1597628

CP006258.1 Saccharomycetaceae sp. 'Ashbya aceri' mitochondrion, complete genome
26996
HG934059.1 Yarrowia lipolytica WSH-Z06 complete genome, chromosome YALIOA
2210998
HG934060.1 Yarrowia lipolytica WSH-Z06 complete genome, chromosome YALIOB
3023969
HG934061.1 Yarrowia lipolytica WSH-Z06 complete genome, chromosome YALIOC
3189950
HG934062.1 Yarrowia lipolytica WSH-Z06 complete genome, chromosome YALIOD
3555057
HG934063.1 Yarrowia lipolytica WSH-Z06 complete genome, chromosome YALIOE
4142257
HG934064.1 Yarrowia lipolytica WSH-Z06 complete genome, chromosome YALIOF
3968352
CP004528.1 Saccharomyces cerevisiae YJM993 plasmid 2 micron, complete sequence
6303
CP006506.1 Saccharomyces cerevisiae YJM993 mitochondrion, complete genome
78907
CM002802.1 Penicillium chrysogenum strain P2niaD18 mitochondrion, complete sequence, whole genome shotgun sequence
27017
CP010980.1 Verticillium dahliae VdLs.17 chromosome 1, complete sequence
5989981
CP010981.1 Verticillium dahliae VdLs.17 chromosome 2, complete sequence
6210300
CP010982.1 Verticillium dahliae VdLs.17 chromosome 3, complete sequence
5894008
CP010983.1 Verticillium dahliae VdLs.17 chromosome 4, complete sequence
4331027
CP010984.1 Verticillium dahliae VdLs.17 chromosome 5, complete sequence
3581864
CP010985.1 Verticillium dahliae VdLs.17 chromosome 6, complete sequence
3402923
CP010986.1 Verticillium dahliae VdLs.17 chromosome 7, complete sequence
3272870
CP010987.1 Verticillium dahliae VdLs.17 chromosome 8, complete sequence
3290897
CP002705.1 Ashbya gossypii FDAG1 chromosome I, complete sequence
693310
CP002706.1 Ashbya gossypii FDAG1 chromosome II, complete sequence
874947
CP002707.1 Ashbya gossypii FDAG1 chromosome III, complete sequence
911446
CP002708.1 Ashbya gossypii FDAG1 chromosome IV, complete sequence
1465717
CP002709.1 Ashbya gossypii FDAG1 chromosome V, complete sequence
1527143
CP002710.1 Ashbya gossypii FDAG1 chromosome VI, complete sequence
1837132
CP002711.1 Ashbya gossypii FDAG1 chromosome VII, complete sequence
1823962
CP002712.1 Ashbya gossypii FDAG1 mitochondrion, complete sequence
23497
CP006476.1 Saccharomyces cerevisiae YJM195 mitochondrion, complete genome
78961
CP004504.1 Saccharomyces cerevisiae YJM244 plasmid 2 micron, complete sequence
6303
CP006477.1 Saccharomyces cerevisiae YJM244 mitochondrion, complete genome
80737
CP004116.1 Saccharomyces cerevisiae YJM1078 plasmid 2micron, complete sequence

6303
CP004115.1 Saccharomyces cerevisiae YJM1078 mitochondrion, complete genome
82025
CP004530.1 Saccharomyces cerevisiae YJM1083 plasmid 2 micron, complete sequence
6118
CP006508.1 Saccharomyces cerevisiae YJM1083 mitochondrion, complete genome
89444
CP004531.1 Saccharomyces cerevisiae YJM1129 plasmid 2 micron, complete sequence
6178
CP006509.1 Saccharomyces cerevisiae YJM1129 mitochondrion, complete genome
81706
CP004502.1 Saccharomyces cerevisiae YJM189 plasmid 2 micron, complete sequence
6178
CP006474.1 Saccharomyces cerevisiae YJM189 mitochondrion, complete genome
80811
CP004503.1 Saccharomyces cerevisiae YJM193 plasmid 2 micron, complete sequence
6303
CP006475.1 Saccharomyces cerevisiae YJM193 mitochondrion, complete genome
84644
CP004505.1 Saccharomyces cerevisiae YJM248 plasmid 2 micron, complete sequence
6178
CP006478.1 Saccharomyces cerevisiae YJM248 mitochondrion, complete genome
86593
CP006479.1 Saccharomyces cerevisiae YJM270 mitochondrion, complete genome
82331
CP004506.1 Saccharomyces cerevisiae YJM271 plasmid 2 micron, complete sequence
6178
CP006480.1 Saccharomyces cerevisiae YJM271 mitochondrion, complete genome
84530
CP004507.1 Saccharomyces cerevisiae YJM320 plasmid 2 micron, complete sequence
6118
CP006481.1 Saccharomyces cerevisiae YJM320 mitochondrion, complete genome
86949
CP004508.1 Saccharomyces cerevisiae YJM326 plasmid 2 micron, complete sequence
6119
CP006482.1 Saccharomyces cerevisiae YJM326 mitochondrion, complete genome
91172
CP004509.1 Saccharomyces cerevisiae YJM428 plasmid 2 micron, complete sequence
6118
CP006483.1 Saccharomyces cerevisiae YJM428 mitochondrion, complete genome
85782
CP004510.1 Saccharomyces cerevisiae YJM450 plasmid 2 micron, complete sequence
6178
CP006484.1 Saccharomyces cerevisiae YJM450 mitochondrion, complete genome
86522
CP004511.1 Saccharomyces cerevisiae YJM451 plasmid 2 micron, complete sequence
6106
CP006485.1 Saccharomyces cerevisiae YJM451 mitochondrion, complete genome
84234
CP004512.1 Saccharomyces cerevisiae YJM453 plasmid 2 micron, complete sequence
6178
CP006486.1 Saccharomyces cerevisiae YJM453 mitochondrion, complete genome
82494
CP004513.1 Saccharomyces cerevisiae YJM456 plasmid 2 micron, complete sequence
6347
CP006487.1 Saccharomyces cerevisiae YJM456 mitochondrion, complete genome
79631
CP006488.1 Saccharomyces cerevisiae YJM470 mitochondrion, complete genome
83571
CP004514.1 Saccharomyces cerevisiae YJM541 plasmid 2 micron, complete sequence

6118
CP006489.1 Saccharomyces cerevisiae YJM541 mitochondrion, complete genome
87003
CP004515.1 Saccharomyces cerevisiae YJM554 plasmid 2 micron, complete sequence
6118
CP006490.1 Saccharomyces cerevisiae YJM554 mitochondrion, complete genome
87008
CP004516.1 Saccharomyces cerevisiae YJM555 plasmid 2 micron, complete sequence
6117
CP006491.1 Saccharomyces cerevisiae YJM555 mitochondrion, complete genome
84193
CP004517.1 Saccharomyces cerevisiae YJM627 plasmid 2 micron, complete sequence
6303
CP006492.1 Saccharomyces cerevisiae YJM627 mitochondrion, complete genome
80791
CP004518.1 Saccharomyces cerevisiae YJM681 plasmid 2 micron, complete sequence
6118
CP006493.1 Saccharomyces cerevisiae YJM681 mitochondrion, complete genome
81045
CP004519.1 Saccharomyces cerevisiae YJM682 plasmid 2 micron, complete sequence
6119
CP006494.1 Saccharomyces cerevisiae YJM682 mitochondrion, complete genome
88887
CP006495.1 Saccharomyces cerevisiae YJM683 mitochondrion, complete genome
88953
CP006496.1 Saccharomyces cerevisiae YJM689 mitochondrion, complete genome
84098
CP006497.1 Saccharomyces cerevisiae YJM693 mitochondrion, complete genome
89161
CP004520.1 Saccharomyces cerevisiae YJM969 plasmid 2 micron, complete sequence
6178
CP006498.1 Saccharomyces cerevisiae YJM969 mitochondrion, complete genome
79066
CP004521.1 Saccharomyces cerevisiae YJM972 plasmid 2 micron, complete sequence
6303
CP006499.1 Saccharomyces cerevisiae YJM972 mitochondrion, complete genome
78907
CP004522.1 Saccharomyces cerevisiae YJM975 plasmid 2 micron, complete sequence
6303
CP006500.1 Saccharomyces cerevisiae YJM975 mitochondrion, complete genome
78950
CP004523.1 Saccharomyces cerevisiae YJM978 plasmid 2 micron, complete sequence
6303
CP006501.1 Saccharomyces cerevisiae YJM978 mitochondrion, complete genome
78916
CP004524.1 Saccharomyces cerevisiae YJM981 plasmid 2 micron, complete sequence
6303
CP006502.1 Saccharomyces cerevisiae YJM981 mitochondrion, complete genome
78916
CP004525.1 Saccharomyces cerevisiae YJM984 plasmid 2 micron, complete sequence
6303
CP006503.1 Saccharomyces cerevisiae YJM984 mitochondrion, complete genome
78907
CP004526.1 Saccharomyces cerevisiae YJM987 plasmid 2 micron, complete sequence
6303
CP006504.1 Saccharomyces cerevisiae YJM987 mitochondrion, complete genome
78974
CP004527.1 Saccharomyces cerevisiae YJM990 plasmid 2 micron, complete sequence
6303
CP006505.1 Saccharomyces cerevisiae YJM990 mitochondrion, complete genome

78916
CP004529.1 Saccharomyces cerevisiae YJM996 plasmid 2 micron, complete sequence
6303
CP006507.1 Saccharomyces cerevisiae YJM996 mitochondrion, complete genome
78907
CP004532.1 Saccharomyces cerevisiae YJM1133 plasmid 2 micron, complete sequence
6118
CP006510.1 Saccharomyces cerevisiae YJM1133 mitochondrion, complete genome
84963
CP004533.1 Saccharomyces cerevisiae YJM1190 plasmid 2 micron, complete sequence
6118
CP006511.2 Saccharomyces cerevisiae YJM1190 mitochondrion, complete genome
88996
CP004534.1 Saccharomyces cerevisiae YJM1199 plasmid 2 micron, complete sequence
6118
CP006512.1 Saccharomyces cerevisiae YJM1199 mitochondrion, complete genome
90633
CP004535.1 Saccharomyces cerevisiae YJM1202 plasmid 2 micron, complete sequence
6118
CP006513.1 Saccharomyces cerevisiae YJM1202 mitochondrion, complete genome
90638
CP004536.1 Saccharomyces cerevisiae YJM1208 plasmid 2 micron, complete sequence
6118
CP006514.1 Saccharomyces cerevisiae YJM1208 mitochondrion, complete genome
84252
CP004537.1 Saccharomyces cerevisiae YJM1242 plasmid 2 micron, complete sequence
6303
CP006515.1 Saccharomyces cerevisiae YJM1242 mitochondrion, complete genome
95658
CP004538.1 Saccharomyces cerevisiae YJM1244 plasmid 2 micron, complete sequence
6303
CP006516.1 Saccharomyces cerevisiae YJM1244 mitochondrion, complete genome
83285
CP006517.1 Saccharomyces cerevisiae YJM1248 mitochondrion, complete genome
81013
CP004539.1 Saccharomyces cerevisiae YJM1250 plasmid 2 micron, complete sequence
6300
CP006518.1 Saccharomyces cerevisiae YJM1250 mitochondrion, complete genome
88746
CP006519.1 Saccharomyces cerevisiae YJM1252 mitochondrion, complete genome
83856
CP006520.1 Saccharomyces cerevisiae YJM1273 mitochondrion, complete genome
77475
CP004540.1 Saccharomyces cerevisiae YJM1304 plasmid 2 micron, complete sequence
6166
CP006521.1 Saccharomyces cerevisiae YJM1304 mitochondrion, complete genome
81083
CP004541.1 Saccharomyces cerevisiae YJM1307 plasmid 2 micron, complete sequence
6178
CP006522.1 Saccharomyces cerevisiae YJM1307 mitochondrion, complete genome
79733
CP004542.1 Saccharomyces cerevisiae YJM1311 plasmid 2 micron, complete sequence
6118
CP006523.1 Saccharomyces cerevisiae YJM1311 mitochondrion, complete genome
87022
CP004543.1 Saccharomyces cerevisiae YJM1326 plasmid 2 micron, complete sequence
6178
CP006524.1 Saccharomyces cerevisiae YJM1326 mitochondrion, complete genome
85887
CP004544.1 Saccharomyces cerevisiae YJM1332 plasmid 2 micron, complete sequence

6303
CP006525.1 Saccharomyces cerevisiae YJM1332 mitochondrion, complete genome
81501
CP004545.1 Saccharomyces cerevisiae YJM1336 plasmid 2 micron, complete sequence
6178
CP006526.1 Saccharomyces cerevisiae YJM1336 mitochondrion, complete genome
82967
CP004546.1 Saccharomyces cerevisiae YJM1338 plasmid 2 micron, complete sequence
6303
CP006527.1 Saccharomyces cerevisiae YJM1338 mitochondrion, complete genome
80970
CP004547.1 Saccharomyces cerevisiae YJM1341 plasmid 2 micron, complete sequence
6178
CP006528.1 Saccharomyces cerevisiae YJM1341 mitochondrion, complete genome
84119
CP006529.1 Saccharomyces cerevisiae YJM1342 mitochondrion, complete genome
85762
CP004548.1 Saccharomyces cerevisiae YJM1355 plasmid 2 micron, complete sequence
6117
CP006530.1 Saccharomyces cerevisiae YJM1355 mitochondrion, complete genome
79418
CP004549.1 Saccharomyces cerevisiae YJM1356 plasmid 2 micron, complete sequence
6303
CP006531.1 Saccharomyces cerevisiae YJM1356 mitochondrion, complete genome
84849
CP004550.1 Saccharomyces cerevisiae YJM1381 plasmid 2 micron, complete sequence
6119
CP006532.1 Saccharomyces cerevisiae YJM1381 mitochondrion, complete genome
83874
CP004551.1 Saccharomyces cerevisiae YJM1383 plasmid 2 micron, complete sequence
6194
CP006533.1 Saccharomyces cerevisiae YJM1383 mitochondrion, complete genome
86820
CP006534.1 Saccharomyces cerevisiae YJM1385 mitochondrion, complete genome
92176
CP004552.1 Saccharomyces cerevisiae YJM1386 plasmid 2 micron, complete sequence
6118
CP006535.1 Saccharomyces cerevisiae YJM1386 mitochondrion, complete genome
85554
CP006536.1 Saccharomyces cerevisiae YJM1387 mitochondrion, complete genome
80666
CP006537.1 Saccharomyces cerevisiae YJM1388 mitochondrion, complete genome
77572
CP006538.1 Saccharomyces cerevisiae YJM1389 mitochondrion, complete genome
80794
CP006539.1 Saccharomyces cerevisiae YJM1399 mitochondrion, complete genome
73450
CP004553.1 Saccharomyces cerevisiae YJM1400 plasmid 2 micron, complete sequence
6094
CP006540.1 Saccharomyces cerevisiae YJM1400 mitochondrion, complete genome
88306
CP004554.1 Saccharomyces cerevisiae YJM1401 plasmid 2 micron, complete sequence
6095
CP006541.1 Saccharomyces cerevisiae YJM1401 mitochondrion, complete genome
81306
CP006542.1 Saccharomyces cerevisiae YJM1402 mitochondrion, complete genome
78309
CP004555.1 Saccharomyces cerevisiae YJM1415 plasmid 2 micron, complete sequence
6178
CP006543.1 Saccharomyces cerevisiae YJM1415 mitochondrion, complete genome

83950
CP004556.1 Saccharomyces cerevisiae YJM1417 plasmid 2 micron, complete sequence
6303
CP006544.1 Saccharomyces cerevisiae YJM1417 mitochondrion, complete genome
82284
CP006545.1 Saccharomyces cerevisiae YJM1418 mitochondrion, complete genome
76794
CP004557.1 Saccharomyces cerevisiae YJM1419 plasmid 2 micron, complete sequence
6118
CP006546.1 Saccharomyces cerevisiae YJM1419 mitochondrion, complete genome
84604
CP004558.1 Saccharomyces cerevisiae YJM1433 plasmid 2 micron, complete sequence
6346
CP006547.1 Saccharomyces cerevisiae YJM1433 mitochondrion, complete genome
81976
CP006548.1 Saccharomyces cerevisiae YJM1434 mitochondrion, complete genome
77515
CP006549.1 Saccharomyces cerevisiae YJM1439 mitochondrion, complete genome
80882
CP006550.1 Saccharomyces cerevisiae YJM1443 mitochondrion, complete genome
77956
CP006551.1 Saccharomyces cerevisiae YJM1444 mitochondrion, complete genome
83736
CP006552.1 Saccharomyces cerevisiae YJM1447 mitochondrion, complete genome
74237
CP004559.1 Saccharomyces cerevisiae YJM1450 plasmid 2 micron, complete sequence
6162
CP006553.1 Saccharomyces cerevisiae YJM1450 mitochondrion, complete genome
76603
CP006554.1 Saccharomyces cerevisiae YJM1460 mitochondrion, complete genome
82773
CP004560.1 Saccharomyces cerevisiae YJM1463 plasmid 2 micron, complete sequence
6179
CP006555.1 Saccharomyces cerevisiae YJM1463 mitochondrion, complete genome
79676
CP004561.1 Saccharomyces cerevisiae YJM1477 plasmid 2 micron, complete sequence
6303
CP006556.1 Saccharomyces cerevisiae YJM1477 mitochondrion, complete genome
79244
CP004562.1 Saccharomyces cerevisiae YJM1478 plasmid 2 micron, complete sequence
6303
CP006557.1 Saccharomyces cerevisiae YJM1478 mitochondrion, complete genome
83765
CP004563.1 Saccharomyces cerevisiae YJM1479 plasmid 2 micron, complete sequence
6094
CP006558.1 Saccharomyces cerevisiae YJM1479 mitochondrion, complete genome
88309
CP004564.1 Saccharomyces cerevisiae YJM1526 plasmid 2 micron, complete sequence
6178
CP006559.1 Saccharomyces cerevisiae YJM1526 mitochondrion, complete genome
81825
CP004565.1 Saccharomyces cerevisiae YJM1527 plasmid 2 micron, complete sequence
6303
CP006560.1 Saccharomyces cerevisiae YJM1527 mitochondrion, complete genome
78592
CP006561.1 Saccharomyces cerevisiae YJM1549 mitochondrion, complete genome
78275
CP006562.1 Saccharomyces cerevisiae YJM1573 mitochondrion, complete genome
78385
CP004566.1 Saccharomyces cerevisiae YJM1574 plasmid 2 micron, complete sequence

6178
CP006563.1 Saccharomyces cerevisiae YJM1574 mitochondrion, complete genome
79923
CP006564.1 Saccharomyces cerevisiae YJM1592 mitochondrion, complete genome
80788
CP004567.1 Saccharomyces cerevisiae YJM1615 plasmid 2 micron, complete sequence
6118
CP006565.1 Saccharomyces cerevisiae YJM1615 mitochondrion, complete genome
84325
CP010913.1 Sporisorium scitamineum strain SSC39 chromosome 1, complete sequence
2009762
CP010914.1 Sporisorium scitamineum strain SSC39 chromosome 2, complete sequence
1671606
CP010915.1 Sporisorium scitamineum strain SSC39 chromosome 3, complete sequence
1566833
CP010916.1 Sporisorium scitamineum strain SSC39 chromosome 4, complete sequence
1271980
CP010917.1 Sporisorium scitamineum strain SSC39 chromosome 5, complete sequence
1055339
CP010918.1 Sporisorium scitamineum strain SSC39 chromosome 6, complete sequence
1032954
CP010919.1 Sporisorium scitamineum strain SSC39 chromosome 7, complete sequence
938453
CP010920.1 Sporisorium scitamineum strain SSC39 chromosome 8, complete sequence
837966
CP010921.1 Sporisorium scitamineum strain SSC39 chromosome 9, complete sequence
808176
CP010922.1 Sporisorium scitamineum strain SSC39 chromosome 10, complete sequence
875830
CP010923.1 Sporisorium scitamineum strain SSC39 chromosome 11, complete sequence
790822
CP010924.1 Sporisorium scitamineum strain SSC39 chromosome 12, complete sequence
716888
CP010925.1 Sporisorium scitamineum strain SSC39 chromosome 13, complete sequence
681008
CP010926.1 Sporisorium scitamineum strain SSC39 chromosome 14, complete sequence
652314
CP010927.1 Sporisorium scitamineum strain SSC39 chromosome 15, complete sequence
634981
CP010928.1 Sporisorium scitamineum strain SSC39 chromosome 16, complete sequence
653278
CP010929.1 Sporisorium scitamineum strain SSC39 chromosome 17, complete sequence
605135
CP010930.1 Sporisorium scitamineum strain SSC39 chromosome 18, complete sequence
598089
CP010931.1 Sporisorium scitamineum strain SSC39 chromosome 19, complete sequence
580969
CP010932.1 Sporisorium scitamineum strain SSC39 chromosome 20, complete sequence
573042
CP010933.1 Sporisorium scitamineum strain SSC39 chromosome 21, complete sequence
539152
CP010934.1 Sporisorium scitamineum strain SSC39 chromosome 22, complete sequence
475902
CP010935.1 Sporisorium scitamineum strain SSC39 chromosome 23, complete sequence
140422
CP010939.1 Sporisorium scitamineum strain SSC39 mitochondrion, complete sequence
88018
CP011547.1 Saccharomyces cerevisiae strain ySR127 chromosome I, complete sequence
224154
CP011548.1 Saccharomyces cerevisiae strain ySR127 chromosome II, complete sequence

808595
CP011549.1 Saccharomyces cerevisiae strain ySR127 chromosome III, complete sequence
313668
CP011550.1 Saccharomyces cerevisiae strain ySR127 chromosome IV, complete sequence
1531682
CP011551.1 Saccharomyces cerevisiae strain ySR127 chromosome V, complete sequence
552153
CP011552.1 Saccharomyces cerevisiae strain ySR127 chromosome VI, complete sequence
270121
CP011553.1 Saccharomyces cerevisiae strain ySR127 chromosome VII, complete sequence
1084888
CP011554.1 Saccharomyces cerevisiae strain ySR127 chromosome VIII, complete sequence
562640
CP011555.1 Saccharomyces cerevisiae strain ySR127 chromosome IX, complete sequence
434899
CP011556.1 Saccharomyces cerevisiae strain ySR127 chromosome X, complete sequence
745071
CP011557.1 Saccharomyces cerevisiae strain ySR127 chromosome XI, complete sequence
666444
CP011558.1 Saccharomyces cerevisiae strain ySR127 chromosome XII, complete sequence
1062294
CP011559.1 Saccharomyces cerevisiae strain ySR127 chromosome XIII, complete sequence
922588
CP011560.1 Saccharomyces cerevisiae strain ySR127 chromosome XIV, complete sequence
784302
CP011561.1 Saccharomyces cerevisiae strain ySR127 chromosome XV, complete sequence
1089058
CP011562.1 Saccharomyces cerevisiae strain ySR127 chromosome XVI, complete sequence
948057
CP011563.1 Saccharomyces cerevisiae strain ySR127 mitochondrion, complete sequence
85731
CM003593.1 Saccharomyces eubayanus strain FM1318 mitochondrion, complete sequence, whole genome shotgun sequence
63999
CP019371.1 Trametes hirsuta strain 072 chromosome 1, complete sequence
4437623
CP019376.1 Trametes hirsuta strain 072 chromosome 3, complete sequence
3674663
CP019377.1 Trametes hirsuta strain 072 chromosome 4, complete sequence
3112363
CP019378.1 Trametes hirsuta strain 072 chromosome 5, complete sequence
3088601
CP019379.1 Trametes hirsuta strain 072 chromosome 6, complete sequence
3045029
CP019380.1 Trametes hirsuta strain 072 chromosome 7, complete sequence
2738869
CP019381.1 Trametes hirsuta strain 072 chromosome 8, complete sequence
2614590
CP019382.1 Trametes hirsuta strain 072 chromosome 9, complete sequence
2611572
CP019370.1 Trametes hirsuta strain 072 chromosome 10, complete sequence
2280792
CP019372.1 Trametes hirsuta strain 072 chromosome 11, complete sequence
2042872
CP019373.1 Trametes hirsuta strain 072 chromosome 12, complete sequence
1949064
CP019374.1 Trametes hirsuta strain 072 chromosome 13, complete sequence
1948569
AP014599.1 Kluyveromyces marxianus DNA, chromosome 1, complete genome, strain: NBRC 1777

1722779
AP014600.1 Kluyveromyces marxianus DNA, chromosome 2, complete genome, strain: NBRC 1777
1688883
AP014601.1 Kluyveromyces marxianus DNA, chromosome 3, complete genome, strain: NBRC 1777
1564943
AP014602.1 Kluyveromyces marxianus DNA, chromosome 4, complete genome, strain: NBRC 1777
1422013
AP014603.1 Kluyveromyces marxianus DNA, chromosome 5, complete genome, strain: NBRC 1777
1351988
AP014604.1 Kluyveromyces marxianus DNA, chromosome 6, complete genome, strain: NBRC 1777
1212614
AP014605.1 Kluyveromyces marxianus DNA, chromosome 7, complete genome, strain: NBRC 1777
940253
AP014606.1 Kluyveromyces marxianus DNA, chromosome 8, complete genome, strain: NBRC 1777
945852
AP014607.1 Kluyveromyces marxianus mitochondrial DNA, complete genome, strain: NBRC 1777
46256
AP012213.1 Kluyveromyces marxianus DMKU3-1042 DNA, complete genome, chromosome 1
1745387
AP012214.1 Kluyveromyces marxianus DMKU3-1042 DNA, complete genome, chromosome 2
1711476
AP012215.1 Kluyveromyces marxianus DMKU3-1042 DNA, complete genome, chromosome 3
1588169
AP012216.1 Kluyveromyces marxianus DMKU3-1042 DNA, complete genome, chromosome 4
1421472
AP012217.1 Kluyveromyces marxianus DMKU3-1042 DNA, complete genome, chromosome 5
1353011
AP012218.1 Kluyveromyces marxianus DMKU3-1042 DNA, complete genome, chromosome 6
1197921
AP012219.1 Kluyveromyces marxianus DMKU3-1042 DNA, complete genome, chromosome 7
963005
AP012220.1 Kluyveromyces marxianus DMKU3-1042 DNA, complete genome, chromosome 8
939718
AP012221.1 Kluyveromyces marxianus DMKU3-1042 mitochondrial DNA, complete genome
46308
CP014242.1 Eremothecium sinecaudum strain ATCC 58844 chromosome II, complete sequence
2061902
CP014243.1 Eremothecium sinecaudum strain ATCC 58844 chromosome III, complete sequence
1398029
CP014244.1 Eremothecium sinecaudum strain ATCC 58844 chromosome IV, complete sequence
1404966
CP014245.1 Eremothecium sinecaudum strain ATCC 58844 chromosome V, complete sequence
1123606
CP014246.1 Eremothecium sinecaudum strain ATCC 58844 chromosome VI, complete sequence
912650
CP014247.1 Eremothecium sinecaudum strain ATCC 58844 chromosome VII, complete sequence
928141
CP014248.1 Eremothecium sinecaudum strain ATCC 58844 chromosome VIII, complete sequence
1093229
CP014249.1 Eremothecium sinecaudum strain ATCC 58844 mitochondrion, complete genome
26238
CP017344.1 Talaromyces pinophilus strain 1-95 chromosome 1, complete sequence
7684667

CP017345.1 Talaromyces pinophilus strain 1-95 chromosome 2, complete sequence
6009755
CP017346.1 Talaromyces pinophilus strain 1-95 chromosome 3, complete sequence
4804168
CP017347.1 Talaromyces pinophilus strain 1-95 chromosome 4, complete sequence
4274674
CP017348.1 Talaromyces pinophilus strain 1-95 chromosome 5, complete sequence
3951088
CP017349.1 Talaromyces pinophilus strain 1-95 chromosome 6, complete sequence
3820271
CP017350.1 Talaromyces pinophilus strain 1-95 chromosome 7, complete sequence
2993891
CP017351.1 Talaromyces pinophilus strain 1-95 chromosome 8, complete sequence
2941929
CP017352.1 Talaromyces pinophilus strain 1-95 mitochondrion, complete genome
31729
CM004311.1 Saccharomyces cerevisiae strain GLBRCY22-3 mitochondrion, complete sequence, whole genome shotgun sequence
82897
CP014501.1 Sugiyamaella lignohabitans strain CBS 10342 chromosome A, complete sequence
5930846
CP014503.1 Sugiyamaella lignohabitans strain CBS 10342 chromosome B, complete sequence
4956242
CP014500.1 Sugiyamaella lignohabitans strain CBS 10342 chromosome C, complete sequence
2773601
CP014502.1 Sugiyamaella lignohabitans strain CBS 10342 chromosome D, complete sequence
2277569
CP015470.1 Agaricus bisporus var. bisporus strain H39 chromosome 1, complete sequence
3779356
CP015471.1 Agaricus bisporus var. bisporus strain H39 chromosome 2, complete sequence
3241356
CP015472.1 Agaricus bisporus var. bisporus strain H39 chromosome 3, complete sequence
3126264
CP015473.1 Agaricus bisporus var. bisporus strain H39 chromosome 4, complete sequence
3115696
CP015474.1 Agaricus bisporus var. bisporus strain H39 chromosome 5, complete sequence
2477729
CP015476.1 Agaricus bisporus var. bisporus strain H39 chromosome 7, complete sequence
2233015
CP015477.1 Agaricus bisporus var. bisporus strain H39 chromosome 8, complete sequence
2155647
CP015479.1 Agaricus bisporus var. bisporus strain H39 chromosome 10, complete sequence
1787784
CP015481.1 Agaricus bisporus var. bisporus strain H39 chromosome 12, complete sequence
1576767
CP015482.1 Agaricus bisporus var. bisporus strain H39 chromosome 13, complete sequence
1405511
CM008299.1 Fusarium oxysporum f. sp. radicis-cucumerinum strain Forc016 mitochondrion, complete sequence, whole genome shotgun sequence
47541
CP014709.1 Komagataella phaffii strain WT chromosome 2, complete sequence
2396783
CP014585.1 Komagataella pastoris strain ATCC 28485 chromosome 2, complete sequence
2687137
CP014716.1 Komagataella phaffii GS115 chromosome 2, complete sequence
2396528
CP017553.1 Yarrowia lipolytica strain CLIB89(W29) chromosome 1A, complete sequence
2257857
CP017554.1 Yarrowia lipolytica strain CLIB89(W29) chromosome 1B, complete sequence
3044971

CP017555.1 Yarrowia lipolytica strain CLIB89(W29) chromosome 1C, complete sequence
3366276
CP017556.1 Yarrowia lipolytica strain CLIB89(W29) chromosome 1D, complete sequence
3629463
CP017557.1 Yarrowia lipolytica strain CLIB89(W29) chromosome 1E, complete sequence
4198534
CP017558.1 Yarrowia lipolytica strain CLIB89(W29) chromosome 1F, complete sequence
4002965
CP017559.1 Yarrowia lipolytica strain CLIB89(W29) mitochondrion, complete genome
47926
CP017814.1 Sclerotinia sclerotiorum chromosome 1, complete sequence
3951982
CP017815.1 Sclerotinia sclerotiorum chromosome 2, complete sequence
3683506
CP017816.1 Sclerotinia sclerotiorum chromosome 3, complete sequence
3351453
CP017817.1 Sclerotinia sclerotiorum chromosome 4, complete sequence
2873318
CP017818.1 Sclerotinia sclerotiorum chromosome 5, complete sequence
2822964
CP017819.1 Sclerotinia sclerotiorum chromosome 6, complete sequence
2483831
CP017821.1 Sclerotinia sclerotiorum chromosome 8, complete sequence
2299506
CP017822.1 Sclerotinia sclerotiorum chromosome 9, complete sequence
2122865
CP017823.1 Sclerotinia sclerotiorum chromosome 10, complete sequence
2105496
CP017824.1 Sclerotinia sclerotiorum chromosome 11, complete sequence
2052242
CP017825.1 Sclerotinia sclerotiorum chromosome 12, complete sequence
1878461
CP017826.1 Sclerotinia sclerotiorum chromosome 13, complete sequence
1845946
CP017827.1 Sclerotinia sclerotiorum chromosome 14, complete sequence
1815632
CP017828.1 Sclerotinia sclerotiorum chromosome 15, complete sequence
1765292
CP017829.1 Sclerotinia sclerotiorum chromosome 16, complete sequence
1419421
CP012809.1 Saccharomycopsis fibuligera x Saccharomycopsis cf. fibuligera strain KJJ81 chromosome A1, complete sequence
4911352
CP012810.1 Saccharomycopsis fibuligera x Saccharomycopsis cf. fibuligera strain KJJ81 chromosome A2, complete sequence
4155011
CP012811.1 Saccharomycopsis fibuligera x Saccharomycopsis cf. fibuligera strain KJJ81 chromosome A3, complete sequence
3014088
CP012812.1 Saccharomycopsis fibuligera x Saccharomycopsis cf. fibuligera strain KJJ81 chromosome A4, complete sequence
2703655
CP012813.1 Saccharomycopsis fibuligera x Saccharomycopsis cf. fibuligera strain KJJ81 chromosome A5, complete sequence
2119959
CP012814.1 Saccharomycopsis fibuligera x Saccharomycopsis cf. fibuligera strain KJJ81 chromosome A6, complete sequence
1448773
CP012815.1 Saccharomycopsis fibuligera x Saccharomycopsis cf. fibuligera strain KJJ81 chromosome A7, complete sequence

1364045
CP012816.1 Saccharomycopsis fibuligera x Saccharomycopsis cf. fibuligera strain KJJ81 chromosome B1, complete sequence
4331628
CP012817.1 Saccharomycopsis fibuligera x Saccharomycopsis cf. fibuligera strain KJJ81 chromosome B2, complete sequence
4036768
CP012818.1 Saccharomycopsis fibuligera x Saccharomycopsis cf. fibuligera strain KJJ81 chromosome B3, complete sequence
2645872
CP012819.1 Saccharomycopsis fibuligera x Saccharomycopsis cf. fibuligera strain KJJ81 chromosome B4, complete sequence
2651701
CP012820.1 Saccharomycopsis fibuligera x Saccharomycopsis cf. fibuligera strain KJJ81 chromosome B5, complete sequence
2379027
CP012821.1 Saccharomycopsis fibuligera x Saccharomycopsis cf. fibuligera strain KJJ81 chromosome B6, complete sequence
1410247
CP012823.1 Saccharomycopsis fibuligera strain KPH12 chromosome 1, complete sequence
4889271
CP012824.1 Saccharomycopsis fibuligera strain KPH12 chromosome 2, complete sequence
4132043
CP012825.1 Saccharomycopsis fibuligera strain KPH12 chromosome 3, complete sequence
3000806
CP012826.1 Saccharomycopsis fibuligera strain KPH12 chromosome 4, complete sequence
2649207
CP012827.1 Saccharomycopsis fibuligera strain KPH12 chromosome 5, complete sequence
2100688
CP012828.1 Saccharomycopsis fibuligera strain KPH12 chromosome 6, complete sequence
1441802
CP012829.1 Saccharomycopsis fibuligera strain KPH12 chromosome 7, complete sequence
1353399
CP015982.1 Saccharomycopsis fibuligera strain ATCC 36309 chromosome 5, complete sequence
2406875
CP015984.1 Saccharomycopsis fibuligera strain ATCC 36309 chromosome 7, complete sequence
1411284
CP019490.1 Zygosaccharomyces parabailii strain ATCC 60483 chromosome 1, complete sequence
2110500
CP019491.1 Zygosaccharomyces parabailii strain ATCC 60483 chromosome 2, complete sequence
2005801
CP019492.1 Zygosaccharomyces parabailii strain ATCC 60483 chromosome 3, complete sequence
1751495
CP019494.1 Zygosaccharomyces parabailii strain ATCC 60483 chromosome 5, complete sequence
1443312
CP019495.1 Zygosaccharomyces parabailii strain ATCC 60483 chromosome 6, complete sequence
1315104
CP019496.1 Zygosaccharomyces parabailii strain ATCC 60483 chromosome 7, complete sequence
1283838
CP019497.1 Zygosaccharomyces parabailii strain ATCC 60483 chromosome 8, complete sequence
1249162
CP019498.1 Zygosaccharomyces parabailii strain ATCC 60483 chromosome 9, complete sequence

1240939
CP019499.1 Zygosaccharomyces parabailii strain ATCC 60483 chromosome 10, complete sequence
1189704
CP019500.1 Zygosaccharomyces parabailii strain ATCC 60483 chromosome 11, complete sequence
1115933
CP019501.1 Zygosaccharomyces parabailii strain ATCC 60483 chromosome 12, complete sequence
1091360
CP019502.1 Zygosaccharomyces parabailii strain ATCC 60483 chromosome 13, complete sequence
1077716
CP019503.1 Zygosaccharomyces parabailii strain ATCC 60483 chromosome 14, complete sequence
1007293
CP019504.1 Zygosaccharomyces parabailii strain ATCC 60483 chromosome 15, complete sequence
858772
CP019505.1 Zygosaccharomyces parabailii strain ATCC 60483 chromosome 16, complete sequence
571967
CP019507.2 Zygosaccharomyces parabailii strain ATCC 60483 plasmid pSB2, complete sequence
5427
CP019506.1 Zygosaccharomyces parabailii strain ATCC 60483 mitochondrion, complete genome
29945
CP016232.1 Trichoderma reesei QM6a chromosome I, complete sequence
6835803
CP016233.1 Trichoderma reesei QM6a chromosome II, complete sequence
6234656
CP016234.1 Trichoderma reesei QM6a chromosome III, complete sequence
5311445
CP016235.1 Trichoderma reesei QM6a chromosome IV, complete sequence
4556834
CP016236.1 Trichoderma reesei QM6a chromosome V, complete sequence
4159965
CP016237.1 Trichoderma reesei QM6a chromosome VI, complete sequence
4000387
CP016238.1 Trichoderma reesei QM6a chromosome VII, complete sequence
3823438
CP020123.1 Saccharomyces cerevisiae strain S288c chromosome I, complete sequence
219929
CP020124.1 Saccharomyces cerevisiae strain S288c chromosome II, complete sequence
813597
CP020125.1 Saccharomyces cerevisiae strain S288c chromosome III, complete sequence
341580
CP020126.1 Saccharomyces cerevisiae strain S288c chromosome IV, complete sequence
1566853
CP020127.1 Saccharomyces cerevisiae strain S288c chromosome V, complete sequence
583092
CP020128.1 Saccharomyces cerevisiae strain S288c chromosome VI, complete sequence
271539
CP020129.1 Saccharomyces cerevisiae strain S288c chromosome VII, complete sequence
1091538
CP020130.1 Saccharomyces cerevisiae strain S288c chromosome VIII, complete sequence
581049
CP020131.1 Saccharomyces cerevisiae strain S288c chromosome IX, complete sequence
440036
CP020132.1 Saccharomyces cerevisiae strain S288c chromosome X, complete sequence
751611

CP020133.1 Saccharomyces cerevisiae strain S288c chromosome XI, complete sequence
666862
CP020135.1 Saccharomyces cerevisiae strain S288c chromosome XIII, complete sequence
930506
CP020136.1 Saccharomyces cerevisiae strain S288c chromosome XIV, complete sequence
777615
CP020137.1 Saccharomyces cerevisiae strain S288c chromosome XV, complete sequence
1091343
CP020138.1 Saccharomyces cerevisiae strain S288c chromosome XVI, complete sequence
954457
CP020139.1 Saccharomyces cerevisiae strain S288c mitochondrion, complete genome
85793
CP020157.1 Saccharomyces cerevisiae strain DBVPG6765 chromosome I, complete sequence
215496
CP020158.1 Saccharomyces cerevisiae strain DBVPG6765 chromosome II, complete sequence
795659
CP020159.1 Saccharomyces cerevisiae strain DBVPG6765 chromosome III, complete sequence
327289
CP020160.1 Saccharomyces cerevisiae strain DBVPG6765 chromosome IV, complete sequence
1488087
CP020161.1 Saccharomyces cerevisiae strain DBVPG6765 chromosome V, complete sequence
576784
CP020162.1 Saccharomyces cerevisiae strain DBVPG6765 chromosome VI, complete sequence
257436
CP020163.1 Saccharomyces cerevisiae strain DBVPG6765 chromosome VII, complete sequence
1070236
CP020164.1 Saccharomyces cerevisiae strain DBVPG6765 chromosome VIII, complete sequence
533397
CP020165.1 Saccharomyces cerevisiae strain DBVPG6765 chromosome IX, complete sequence
419821
CP020166.1 Saccharomyces cerevisiae strain DBVPG6765 chromosome X, complete sequence
730957
CP020167.1 Saccharomyces cerevisiae strain DBVPG6765 chromosome XI, complete sequence
657417
CP020169.1 Saccharomyces cerevisiae strain DBVPG6765 chromosome XIII, complete sequence
913017
CP020170.1 Saccharomyces cerevisiae strain DBVPG6765 chromosome XIV, complete sequence
765110
CP020171.1 Saccharomyces cerevisiae strain DBVPG6765 chromosome XV, complete sequence
1120088
CP020172.1 Saccharomyces cerevisiae strain DBVPG6765 chromosome XVI, complete sequence
920208
CP020173.1 Saccharomyces cerevisiae strain DBVPG6765 mitochondrion, complete genome
81722
CP020174.1 Saccharomyces cerevisiae strain SK1 chromosome I, complete sequence
228861
CP020175.1 Saccharomyces cerevisiae strain SK1 chromosome II, complete sequence
829469
CP020176.1 Saccharomyces cerevisiae strain SK1 chromosome III, complete sequence
340914
CP020177.1 Saccharomyces cerevisiae strain SK1 chromosome IV, complete sequence
1486921
CP020178.1 Saccharomyces cerevisiae strain SK1 chromosome V, complete sequence
589812
CP020179.1 Saccharomyces cerevisiae strain SK1 chromosome VI, complete sequence
299318
CP020180.1 Saccharomyces cerevisiae strain SK1 chromosome VII, complete sequence
1080440
CP020181.1 Saccharomyces cerevisiae strain SK1 chromosome VIII, complete sequence
542723

CP020182.1 Saccharomyces cerevisiae strain SK1 chromosome IX, complete sequence
449612
CP020183.1 Saccharomyces cerevisiae strain SK1 chromosome X, complete sequence
753937
CP020184.1 Saccharomyces cerevisiae strain SK1 chromosome XI, complete sequence
690901
CP020186.1 Saccharomyces cerevisiae strain SK1 chromosome XIII, complete sequence
923535
CP020187.1 Saccharomyces cerevisiae strain SK1 chromosome XIV, complete sequence
791982
CP020188.1 Saccharomyces cerevisiae strain SK1 chromosome XV, complete sequence
1053869
CP020189.1 Saccharomyces cerevisiae strain SK1 chromosome XVI, complete sequence
946846
CP020190.1 Saccharomyces cerevisiae strain SK1 mitochondrion, complete genome
84638
CP020208.1 Saccharomyces cerevisiae strain YPS128 chromosome I, complete sequence
237661
CP020209.1 Saccharomyces cerevisiae strain YPS128 chromosome II, complete sequence
808481
CP020210.1 Saccharomyces cerevisiae strain YPS128 chromosome III, complete sequence
319304
CP020211.1 Saccharomyces cerevisiae strain YPS128 chromosome IV, complete sequence
1495051
CP020212.1 Saccharomyces cerevisiae strain YPS128 chromosome V, complete sequence
575962
CP020213.1 Saccharomyces cerevisiae strain YPS128 chromosome VI, complete sequence
289276
CP020214.1 Saccharomyces cerevisiae strain YPS128 chromosome VII, complete sequence
1082279
CP020215.1 Saccharomyces cerevisiae strain YPS128 chromosome VIII, complete sequence
543240
CP020216.1 Saccharomyces cerevisiae strain YPS128 chromosome IX, complete sequence
440919
CP020217.1 Saccharomyces cerevisiae strain YPS128 chromosome X, complete sequence
723414
CP020218.1 Saccharomyces cerevisiae strain YPS128 chromosome XI, complete sequence
667446
CP020220.1 Saccharomyces cerevisiae strain YPS128 chromosome XIII, complete sequence
928527
CP020221.1 Saccharomyces cerevisiae strain YPS128 chromosome XIV, complete sequence
766731
CP020222.1 Saccharomyces cerevisiae strain YPS128 chromosome XV, complete sequence
1072763
CP020223.1 Saccharomyces cerevisiae strain YPS128 chromosome XVI, complete sequence
932263
CP020224.1 Saccharomyces cerevisiae strain YPS128 mitochondrion, complete genome
77479
CP020225.1 Saccharomyces cerevisiae strain UWOPS03-461.4 chromosome I, complete sequence
214332
CP020226.1 Saccharomyces cerevisiae strain UWOPS03-461.4 chromosome II, complete sequence
815151
CP020227.1 Saccharomyces cerevisiae strain UWOPS03-461.4 chromosome III, complete sequence
309137
CP020228.1 Saccharomyces cerevisiae strain UWOPS03-461.4 chromosome IV, complete sequence
1468232

CP020229.1 Saccharomyces cerevisiae strain UWOPS03-461.4 chromosome V, complete sequence
555692
CP020230.1 Saccharomyces cerevisiae strain UWOPS03-461.4 chromosome VI, complete sequence
290867
CP020231.1 Saccharomyces cerevisiae strain UWOPS03-461.4 chromosome VII, complete sequence
632616
CP020232.1 Saccharomyces cerevisiae strain UWOPS03-461.4 chromosome VIII, complete sequence
738767
CP020233.1 Saccharomyces cerevisiae strain UWOPS03-461.4 chromosome IX, complete sequence
428968
CP020234.1 Saccharomyces cerevisiae strain UWOPS03-461.4 chromosome X, complete sequence
1092164
CP020235.1 Saccharomyces cerevisiae strain UWOPS03-461.4 chromosome XI, complete sequence
792116
CP020237.1 Saccharomyces cerevisiae strain UWOPS03-461.4 chromosome XIII, complete sequence
662343
CP020238.1 Saccharomyces cerevisiae strain UWOPS03-461.4 chromosome XIV, complete sequence
765143
CP020239.1 Saccharomyces cerevisiae strain UWOPS03-461.4 chromosome XV, complete sequence
1062502
CP020240.1 Saccharomyces cerevisiae strain UWOPS03-461.4 chromosome XVI, complete sequence
909189
CP020241.1 Saccharomyces cerevisiae strain UWOPS03-461.4 mitochondrion, complete genome
74179
CP020191.1 Saccharomyces cerevisiae strain Y12 chromosome I, complete sequence
197190
CP020192.1 Saccharomyces cerevisiae strain Y12 chromosome II, complete sequence
800619
CP020193.1 Saccharomyces cerevisiae strain Y12 chromosome III, complete sequence
322503
CP020194.1 Saccharomyces cerevisiae strain Y12 chromosome IV, complete sequence
1497473
CP020195.1 Saccharomyces cerevisiae strain Y12 chromosome V, complete sequence
575802
CP020196.1 Saccharomyces cerevisiae strain Y12 chromosome VI, complete sequence
285938
CP020197.1 Saccharomyces cerevisiae strain Y12 chromosome VII, complete sequence
1107723
CP020198.1 Saccharomyces cerevisiae strain Y12 chromosome VIII, complete sequence
547529
CP020199.1 Saccharomyces cerevisiae strain Y12 chromosome IX, complete sequence
431184
CP020200.1 Saccharomyces cerevisiae strain Y12 chromosome X, complete sequence
725652
CP020201.1 Saccharomyces cerevisiae strain Y12 chromosome XI, complete sequence
692235
CP020203.1 Saccharomyces cerevisiae strain Y12 chromosome XIII, complete sequence
903361
CP020204.1 Saccharomyces cerevisiae strain Y12 chromosome XIV, complete sequence

800685
CP020205.1 Saccharomyces cerevisiae strain Y12 chromosome XV, complete sequence
1048295
CP020206.1 Saccharomyces cerevisiae strain Y12 chromosome XVI, complete sequence
901780
CP020207.1 Saccharomyces cerevisiae strain Y12 mitochondrion, complete genome
82868
CP020140.1 Saccharomyces cerevisiae strain DBVPG6044 chromosome I, complete sequence
217365
CP020141.1 Saccharomyces cerevisiae strain DBVPG6044 chromosome II, complete sequence
815565
CP020142.1 Saccharomyces cerevisiae strain DBVPG6044 chromosome III, complete sequence
332771
CP020143.1 Saccharomyces cerevisiae strain DBVPG6044 chromosome IV, complete sequence
1486873
CP020144.1 Saccharomyces cerevisiae strain DBVPG6044 chromosome V, complete sequence
572248
CP020145.1 Saccharomyces cerevisiae strain DBVPG6044 chromosome VI, complete sequence
298678
CP020146.1 Saccharomyces cerevisiae strain DBVPG6044 chromosome VII, complete sequence
1078455
CP020147.1 Saccharomyces cerevisiae strain DBVPG6044 chromosome VIII, complete sequence
535633
CP020148.1 Saccharomyces cerevisiae strain DBVPG6044 chromosome IX, complete sequence
443501
CP020149.1 Saccharomyces cerevisiae strain DBVPG6044 chromosome X, complete sequence
728645
CP020150.1 Saccharomyces cerevisiae strain DBVPG6044 chromosome XI, complete sequence
695907
CP020152.1 Saccharomyces cerevisiae strain DBVPG6044 chromosome XIII, complete sequence
930256
CP020153.1 Saccharomyces cerevisiae strain DBVPG6044 chromosome XIV, complete sequence
784343
CP020154.1 Saccharomyces cerevisiae strain DBVPG6044 chromosome XV, complete sequence
1062383
CP020155.1 Saccharomyces cerevisiae strain DBVPG6044 chromosome XVI, complete sequence
939911
CP020156.1 Saccharomyces cerevisiae strain DBVPG6044 mitochondrion, complete genome
81093
CP020242.1 Saccharomyces paradoxus strain CBS432 chromosome I, complete sequence
235665
CP020243.1 Saccharomyces paradoxus strain CBS432 chromosome II, complete sequence
835812
CP020244.1 Saccharomyces paradoxus strain CBS432 chromosome III, complete sequence
357685
CP020245.1 Saccharomyces paradoxus strain CBS432 chromosome IV, complete sequence
1477988
CP020246.1 Saccharomyces paradoxus strain CBS432 chromosome V, complete sequence
565208
CP020247.1 Saccharomyces paradoxus strain CBS432 chromosome VI, complete sequence
296034
CP020248.1 Saccharomyces paradoxus strain CBS432 chromosome VII, complete sequence
1119193
CP020249.1 Saccharomyces paradoxus strain CBS432 chromosome VIII, complete sequence
531267
CP020250.1 Saccharomyces paradoxus strain CBS432 chromosome IX, complete sequence
443128
CP020251.1 Saccharomyces paradoxus strain CBS432 chromosome X, complete sequence
743843
CP020252.1 Saccharomyces paradoxus strain CBS432 chromosome XI, complete sequence

690553
CP020254.1 Saccharomyces paradoxus strain CBS432 chromosome XIII, complete sequence
941477
CP020255.1 Saccharomyces paradoxus strain CBS432 chromosome XIV, complete sequence
778926
CP020256.1 Saccharomyces paradoxus strain CBS432 chromosome XV, complete sequence
1078405
CP020257.1 Saccharomyces paradoxus strain CBS432 chromosome XVI, complete sequence
903028
CP020258.1 Saccharomyces paradoxus strain CBS432 mitochondrion, complete genome
71482
CP020259.1 Saccharomyces paradoxus strain N44 chromosome I, complete sequence
224181
CP020260.1 Saccharomyces paradoxus strain N44 chromosome II, complete sequence
800085
CP020261.1 Saccharomyces paradoxus strain N44 chromosome III, complete sequence
312241
CP020262.1 Saccharomyces paradoxus strain N44 chromosome IV, complete sequence
1466838
CP020263.1 Saccharomyces paradoxus strain N44 chromosome V, complete sequence
553973
CP020264.1 Saccharomyces paradoxus strain N44 chromosome VI, complete sequence
296174
CP020265.1 Saccharomyces paradoxus strain N44 chromosome VII, complete sequence
1082435
CP020266.1 Saccharomyces paradoxus strain N44 chromosome VIII, complete sequence
529296
CP020267.1 Saccharomyces paradoxus strain N44 chromosome IX, complete sequence
442403
CP020268.1 Saccharomyces paradoxus strain N44 chromosome X, complete sequence
729272
CP020269.1 Saccharomyces paradoxus strain N44 chromosome XI, complete sequence
679893
CP020271.1 Saccharomyces paradoxus strain N44 chromosome XIII, complete sequence
926729
CP020272.1 Saccharomyces paradoxus strain N44 chromosome XIV, complete sequence
776644
CP020273.1 Saccharomyces paradoxus strain N44 chromosome XV, complete sequence
1061158
CP020274.1 Saccharomyces paradoxus strain N44 chromosome XVI, complete sequence
904322
CP020275.1 Saccharomyces paradoxus strain N44 mitochondrion, complete genome
69948
CP020276.1 Saccharomyces paradoxus strain YPS138 chromosome I, complete sequence
254647
CP020277.1 Saccharomyces paradoxus strain YPS138 chromosome II, complete sequence
789597
CP020278.1 Saccharomyces paradoxus strain YPS138 chromosome III, complete sequence
315836
CP020279.1 Saccharomyces paradoxus strain YPS138 chromosome IV, complete sequence
1469832
CP020280.1 Saccharomyces paradoxus strain YPS138 chromosome V, complete sequence
571131
CP020281.1 Saccharomyces paradoxus strain YPS138 chromosome VI, complete sequence
296071
CP020282.1 Saccharomyces paradoxus strain YPS138 chromosome VII, complete sequence
1067218
CP020283.1 Saccharomyces paradoxus strain YPS138 chromosome VIII, complete sequence
531566
CP020284.1 Saccharomyces paradoxus strain YPS138 chromosome IX, complete sequence

431692
CP020285.1 Saccharomyces paradoxus strain YPS138 chromosome X, complete sequence
707288
CP020286.1 Saccharomyces paradoxus strain YPS138 chromosome XI, complete sequence
687600
CP020288.1 Saccharomyces paradoxus strain YPS138 chromosome XIII, complete sequence
935281
CP020289.1 Saccharomyces paradoxus strain YPS138 chromosome XIV, complete sequence
769158
CP020290.1 Saccharomyces paradoxus strain YPS138 chromosome XV, complete sequence
1049886
CP020291.1 Saccharomyces paradoxus strain YPS138 chromosome XVI, complete sequence
905611
CP020292.1 Saccharomyces paradoxus strain YPS138 mitochondrion, complete genome
71396
CP020293.1 Saccharomyces paradoxus strain UFRJ50816 chromosome I, complete sequence
220177
CP020294.1 Saccharomyces paradoxus strain UFRJ50816 chromosome II, complete sequence
731301
CP020295.1 Saccharomyces paradoxus strain UFRJ50816 chromosome III, complete sequence
308064
CP020296.1 Saccharomyces paradoxus strain UFRJ50816 chromosome IV, complete sequence
1391618
CP020297.1 Saccharomyces paradoxus strain UFRJ50816 chromosome V, complete sequence
577572
CP020298.1 Saccharomyces paradoxus strain UFRJ50816 chromosome VI, complete sequence
282957
CP020299.1 Saccharomyces paradoxus strain UFRJ50816 chromosome VII, complete sequence
1105967
CP020300.1 Saccharomyces paradoxus strain UFRJ50816 chromosome VIII, complete sequence
546662
CP020301.1 Saccharomyces paradoxus strain UFRJ50816 chromosome IX, complete sequence
374625
CP020302.1 Saccharomyces paradoxus strain UFRJ50816 chromosome X, complete sequence
751133
CP020303.1 Saccharomyces paradoxus strain UFRJ50816 chromosome XI, complete sequence
805821
CP020305.1 Saccharomyces paradoxus strain UFRJ50816 chromosome XIII, complete sequence
869764
CP020306.1 Saccharomyces paradoxus strain UFRJ50816 chromosome XIV, complete sequence
872155
CP020307.1 Saccharomyces paradoxus strain UFRJ50816 chromosome XV, complete sequence
1201643
CP020308.1 Saccharomyces paradoxus strain UFRJ50816 chromosome XVI, complete sequence
1029411
CP020309.1 Saccharomyces paradoxus strain UFRJ50816 mitochondrion, complete genome
77386
CP020310.1 Saccharomyces paradoxus strain UWOPS91-917.1 chromosome I, complete sequence
211120
CP020311.1 Saccharomyces paradoxus strain UWOPS91-917.1 chromosome II, complete sequence
801843
CP020312.1 Saccharomyces paradoxus strain UWOPS91-917.1 chromosome III, complete sequence
316051
CP020313.1 Saccharomyces paradoxus strain UWOPS91-917.1 chromosome IV, complete sequence
1513677

CP020314.1 Saccharomyces paradoxus strain UWOPS91-917.1 chromosome V, complete sequence
1007496
CP020315.1 Saccharomyces paradoxus strain UWOPS91-917.1 chromosome VI, complete sequence
306036
CP020316.1 Saccharomyces paradoxus strain UWOPS91-917.1 chromosome VII, complete sequence
1057931
CP020317.1 Saccharomyces paradoxus strain UWOPS91-917.1 chromosome VIII, complete sequence
526907
CP020318.1 Saccharomyces paradoxus strain UWOPS91-917.1 chromosome IX, complete sequence
439147
CP020319.1 Saccharomyces paradoxus strain UWOPS91-917.1 chromosome X, complete sequence
750185
CP020320.1 Saccharomyces paradoxus strain UWOPS91-917.1 chromosome XI, complete sequence
681961
CP020322.1 Saccharomyces paradoxus strain UWOPS91-917.1 chromosome XIII, complete sequence
475121
CP020323.1 Saccharomyces paradoxus strain UWOPS91-917.1 chromosome XIV, complete sequence
769517
CP020324.1 Saccharomyces paradoxus strain UWOPS91-917.1 chromosome XV, complete sequence
1052871
CP020325.1 Saccharomyces paradoxus strain UWOPS91-917.1 chromosome XVI, complete sequence
905577
CP020326.1 Saccharomyces paradoxus strain UWOPS91-917.1 mitochondrion, complete genome
73171
CP021239.1 Kluyveromyces lactis strain GG799 chromosome A, complete sequence
1050866
CP021240.1 Kluyveromyces lactis strain GG799 chromosome B, complete sequence
1319333
CP021241.1 Kluyveromyces lactis strain GG799 chromosome C, complete sequence
1752996
CP021242.1 Kluyveromyces lactis strain GG799 chromosome D, complete sequence
1700866
CP021243.1 Kluyveromyces lactis strain GG799 chromosome E, complete sequence
2217437
CP021244.1 Kluyveromyces lactis strain GG799 chromosome F, complete sequence
2601250
CP021245.1 Kluyveromyces lactis strain GG799 mitochondrion, complete genome
39914
CM007980.1 Saccharomyces cerevisiae W303 plasmid p2-micron, complete sequence, whole genome shotgun sequence
6300
CP022321.1 Cryptococcus neoformans var. grubii chromosome 1, complete sequence
2291500
CP022322.1 Cryptococcus neoformans var. grubii chromosome 2, complete sequence
1621676
CP022323.1 Cryptococcus neoformans var. grubii chromosome 3, complete sequence
1574972
CP022324.1 Cryptococcus neoformans var. grubii chromosome 4, complete sequence
1084805

CP022325.1 Cryptococcus neoformans var. grubii chromosome 5, complete sequence
1814975
CP022326.1 Cryptococcus neoformans var. grubii chromosome 6, complete sequence
1422463
CP022327.1 Cryptococcus neoformans var. grubii chromosome 7, complete sequence
1399209
CP022328.1 Cryptococcus neoformans var. grubii chromosome 8, complete sequence
1398693
CP022329.1 Cryptococcus neoformans var. grubii chromosome 9, complete sequence
1186813
CP022330.1 Cryptococcus neoformans var. grubii chromosome 10, complete sequence
1059962
CP022331.1 Cryptococcus neoformans var. grubii chromosome 11, complete sequence
1562107
CP022332.1 Cryptococcus neoformans var. grubii chromosome 12, complete sequence
774060
CP022333.1 Cryptococcus neoformans var. grubii chromosome 13, complete sequence
756017
CP022334.1 Cryptococcus neoformans var. grubii chromosome 14, complete sequence
942472
CP022335.1 Cryptococcus neoformans var. grubii strain KN99 mitochondrion, complete genome
24923
CM008094.1 [Candida] glabrata strain DSY562 mitochondrion, complete sequence, whole genome shotgun sequence
20086
CM008107.1 [Candida] glabrata strain DSY565 mitochondrion, complete sequence, whole genome shotgun sequence
20086
CP022800.1 Parastagonospora nodorum isolate LDN03-Sn4 chromosome 1, complete sequence
3532264
CP022801.1 Parastagonospora nodorum isolate LDN03-Sn4 chromosome 2, complete sequence
2872662
CP022803.1 Parastagonospora nodorum isolate LDN03-Sn4 chromosome 3, complete sequence
2401307
CP022802.1 Parastagonospora nodorum isolate LDN03-Sn4 chromosome 4, complete sequence
2335616
CP022804.1 Parastagonospora nodorum isolate LDN03-Sn4 chromosome 5, complete sequence
2001616
CP022806.1 Parastagonospora nodorum isolate LDN03-Sn4 chromosome 6, complete sequence
1779389
CP022807.1 Parastagonospora nodorum isolate LDN03-Sn4 chromosome 7, complete sequence
1731349
CP022808.1 Parastagonospora nodorum isolate LDN03-Sn4 chromosome 8, complete sequence
1712619
CP022809.1 Parastagonospora nodorum isolate LDN03-Sn4 chromosome 9, complete sequence
1657153
CP022805.1 Parastagonospora nodorum isolate LDN03-Sn4 chromosome 10, complete sequence
1651622
CP022812.1 Parastagonospora nodorum isolate LDN03-Sn4 chromosome 11, complete sequence
1479508
CP022811.1 Parastagonospora nodorum isolate LDN03-Sn4 chromosome 13, complete sequence
1441076
CP022813.1 Parastagonospora nodorum isolate LDN03-Sn4 chromosome 14, complete sequence
1377913
CP022814.1 Parastagonospora nodorum isolate LDN03-Sn4 chromosome 15, complete sequence
1346229
CP022816.1 Parastagonospora nodorum isolate LDN03-Sn4 chromosome 16, complete sequence
1292678
CP022815.1 Parastagonospora nodorum isolate LDN03-Sn4 chromosome 17, complete sequence
1279747

CP022817.1 Parastagonospora nodorum isolate LDN03-Sn4 chromosome 18, complete sequence
1274223
CP022818.1 Parastagonospora nodorum isolate LDN03-Sn4 chromosome 19, complete sequence
1125282
CP022820.1 Parastagonospora nodorum isolate LDN03-Sn4 chromosome 20, complete sequence
1090035
CP022819.1 Parastagonospora nodorum isolate LDN03-Sn4 chromosome 21, complete sequence
1077963
CP022822.1 Parastagonospora nodorum isolate LDN03-Sn4 chromosome 23, complete sequence
476058
CP022852.1 Parastagonospora nodorum isolate Sn79-1087 chromosome 1, complete sequence
3434512
CP022853.1 Parastagonospora nodorum isolate Sn79-1087 chromosome 2, complete sequence
2797422
CP022855.1 Parastagonospora nodorum isolate Sn79-1087 chromosome 3, complete sequence
2253675
CP022854.1 Parastagonospora nodorum isolate Sn79-1087 chromosome 4, complete sequence
2256902
CP022856.1 Parastagonospora nodorum isolate Sn79-1087 chromosome 5, complete sequence
1872577
CP022858.1 Parastagonospora nodorum isolate Sn79-1087 chromosome 6, complete sequence
1730842
CP022857.1 Parastagonospora nodorum isolate Sn79-1087 chromosome 7, complete sequence
1705187
CP022860.1 Parastagonospora nodorum isolate Sn79-1087 chromosome 8, complete sequence
1558419
CP022859.1 Parastagonospora nodorum isolate Sn79-1087 chromosome 9, complete sequence
1583228
CP022869.1 Parastagonospora nodorum isolate Sn79-1087 chromosome 10, complete sequence
1130272
CP022861.1 Parastagonospora nodorum isolate Sn79-1087 chromosome 11, complete sequence
1388164
CP022862.1 Parastagonospora nodorum isolate Sn79-1087 chromosome 13, complete sequence
1396077
CP022865.1 Parastagonospora nodorum isolate Sn79-1087 chromosome 14, complete sequence
1299335
CP022864.1 Parastagonospora nodorum isolate Sn79-1087 chromosome 15, complete sequence
1255146
CP022868.1 Parastagonospora nodorum isolate Sn79-1087 chromosome 16, complete sequence
1170298
CP022866.1 Parastagonospora nodorum isolate Sn79-1087 chromosome 17, complete sequence
1211780
CP022873.1 Parastagonospora nodorum isolate Sn79-1087 chromosome 19, complete sequence
995738
CP022872.1 Parastagonospora nodorum isolate Sn79-1087 chromosome 20, complete sequence
1048157
CP022871.1 Parastagonospora nodorum isolate Sn79-1087 chromosome 21, complete sequence
1070356
CP022867.1 Parastagonospora nodorum isolate Sn79-1087 chromosome 22, complete sequence
1168050
CM008263.1 Phellinus noxius strain FFPRI411160 mitochondrion, complete sequence, whole genome shotgun sequence
163449
CP023456.1 Kluyveromyces marxianus strain NRRL Y-6860 chromosome 1, complete sequence
1734405
CP023457.1 Kluyveromyces marxianus strain NRRL Y-6860 chromosome 2, complete sequence
1699062
CP023458.1 Kluyveromyces marxianus strain NRRL Y-6860 chromosome 3, complete sequence
1559681
CP023459.1 Kluyveromyces marxianus strain NRRL Y-6860 chromosome 4, complete sequence

1406771
CP023460.1 Kluyveromyces marxianus strain NRRL Y-6860 chromosome 5, complete sequence
1373102
CP023461.1 Kluyveromyces marxianus strain NRRL Y-6860 chromosome 6, complete sequence
1205333
CP023462.1 Kluyveromyces marxianus strain NRRL Y-6860 chromosome 7, complete sequence
928821
CP023463.1 Kluyveromyces marxianus strain NRRL Y-6860 chromosome 8, complete sequence
930443
CM008875.1 Saccharomyces cerevisiae strain CEN.PK113-7D plasmid p2uM, complete sequence, whole genome shotgun sequence
6318
CM008876.1 Saccharomyces cerevisiae strain CEN.PK113-7D mitochondrion, complete sequence, whole genome shotgun sequence
86616
CP022024.1 Alternaria solani isolate NL03003 chromosome 1, complete sequence
6940169
CP022025.1 Alternaria solani isolate NL03003 chromosome 2, complete sequence
5062150
CP022026.1 Alternaria solani isolate NL03003 chromosome 3, complete sequence
3306264
CP022027.1 Alternaria solani isolate NL03003 chromosome 4, complete sequence
2866555
CP022028.1 Alternaria solani isolate NL03003 chromosome 5, complete sequence
2771896
CP022029.1 Alternaria solani isolate NL03003 chromosome 6, complete sequence
2590027
CP022030.1 Alternaria solani isolate NL03003 chromosome 7, complete sequence
2546252
CP022031.1 Alternaria solani isolate NL03003 chromosome 8, complete sequence
2508373
CP022032.1 Alternaria solani isolate NL03003 chromosome 9, complete sequence
2309181
CP022033.1 Alternaria solani isolate NL03003 chromosome 10, complete sequence
1878275
CP025759.1 Cryptococcus gattii VGII R265 chromosome 1, complete sequence
2207848
CP025760.1 Cryptococcus gattii VGII R265 chromosome 2, complete sequence
2171391
CP025761.1 Cryptococcus gattii VGII R265 chromosome 3, complete sequence
1968033
CP025762.1 Cryptococcus gattii VGII R265 chromosome 4, complete sequence
1750766
CP025763.1 Cryptococcus gattii VGII R265 chromosome 5, complete sequence
1316417
CP025764.1 Cryptococcus gattii VGII R265 chromosome 6, complete sequence
1307262
CP025765.1 Cryptococcus gattii VGII R265 chromosome 7, complete sequence
1243496
CP025766.1 Cryptococcus gattii VGII R265 chromosome 8, complete sequence
1233638
CP025767.1 Cryptococcus gattii VGII R265 chromosome 9, complete sequence
972356
CP025768.1 Cryptococcus gattii VGII R265 chromosome 10, complete sequence
815166
CP025769.1 Cryptococcus gattii VGII R265 chromosome 11, complete sequence
733013
CP025770.1 Cryptococcus gattii VGII R265 chromosome 12, complete sequence
663894
CP025771.1 Cryptococcus gattii VGII R265 chromosome 13, complete sequence

650339
CP025772.1 Cryptococcus gattii VGII R265 chromosome 14, complete sequence
516503
CP025773.1 Cryptococcus gattii VGII R265 mitochondrion, complete genome
31189
CP027647.1 Torulaspora delbrueckii strain COFT1 chromosome 1, complete sequence
1420397
CP027648.1 Torulaspora delbrueckii strain COFT1 chromosome 2, complete sequence
1398547
CP027649.1 Torulaspora delbrueckii strain COFT1 chromosome 3, complete sequence
1318504
CP027650.1 Torulaspora delbrueckii strain COFT1 chromosome 4, complete sequence
1238785
CP027651.1 Torulaspora delbrueckii strain COFT1 chromosome 5, complete sequence
1073930
CP027652.1 Torulaspora delbrueckii strain COFT1 chromosome 6, complete sequence
1067747
CP027653.1 Torulaspora delbrueckii strain COFT1 chromosome 7, complete sequence
927760
CP027654.1 Torulaspora delbrueckii strain COFT1 chromosome 8, complete sequence
863982
CP027655.1 Torulaspora delbrueckii strain COFT1 mitochondrion, complete genome
47174
CP021098.1 Pichia kudriavzevii strain SJP mitochondrion, complete genome
51126
CP028531.1 Pichia kudriavzevii strain CBS5147 chromosome 1, complete sequence
2861343
CP028532.1 Pichia kudriavzevii strain CBS5147 chromosome 2, complete sequence
2715831
CP028533.1 Pichia kudriavzevii strain CBS5147 chromosome 3, complete sequence
2541826
CP028534.1 Pichia kudriavzevii strain CBS5147 chromosome 4, complete sequence
1374539
CP028535.1 Pichia kudriavzevii strain CBS5147 chromosome 5, complete sequence
1289534
CP028773.1 Pichia kudriavzevii strain CBS573 chromosome 1, complete sequence
2852272
CP028774.1 Pichia kudriavzevii strain CBS573 chromosome 2, complete sequence
2745854
CP028775.1 Pichia kudriavzevii strain CBS573 chromosome 3, complete sequence
2541747
CP028776.1 Pichia kudriavzevii strain CBS573 chromosome 4, complete sequence
1383544
CP028777.1 Pichia kudriavzevii strain CBS573 chromosome 5, complete sequence
1289138
CP028778.1 Pichia kudriavzevii strain CBS573 mitochondrion, complete genome
51340
CP026301.1 Saccharomyces cerevisiae strain BY4742 chromosome I, complete sequence
230490
CP026296.1 Saccharomyces cerevisiae strain BY4742 chromosome II, complete sequence
822297
CP026297.1 Saccharomyces cerevisiae strain BY4742 chromosome III, complete sequence
333764
CP026298.1 Saccharomyces cerevisiae strain BY4742 chromosome IV, complete sequence
1545606
CP026299.1 Saccharomyces cerevisiae strain BY4742 chromosome V, complete sequence
582048
CP026302.1 Saccharomyces cerevisiae strain BY4742 chromosome VI, complete sequence
270887
CP026294.1 Saccharomyces cerevisiae strain BY4742 chromosome VII, complete sequence

1091388
CP026287.1 Saccharomyces cerevisiae strain BY4742 chromosome VIII, complete sequence
576806
CP026295.1 Saccharomyces cerevisiae strain BY4742 chromosome IX, complete sequence
440032
CP026288.1 Saccharomyces cerevisiae strain BY4742 chromosome X, complete sequence
745649
CP026289.1 Saccharomyces cerevisiae strain BY4742 chromosome XI, complete sequence
666876
CP026300.1 Saccharomyces cerevisiae strain BY4742 chromosome XII, complete sequence
1104511
CP026291.1 Saccharomyces cerevisiae strain BY4742 chromosome XIII, complete sequence
930523
CP026293.1 Saccharomyces cerevisiae strain BY4742 chromosome XIV, complete sequence
784585
CP026303.1 Saccharomyces cerevisiae strain BY4742 chromosome XV, complete sequence
1091321
CP026290.1 Saccharomyces cerevisiae strain BY4742 chromosome XVI, complete sequence
948685
HG970332.2 Fusarium graminearum chromosome 1, complete genome
11760891
HG970333.1 Fusarium graminearum chromosome 2, complete genome
8997558
HG970334.1 Fusarium graminearum chromosome 3, complete genome
7792947
HG970335.2 Fusarium graminearum chromosome 4, complete genome
9395062
HG970331.1 Fusarium graminearum mitochondrial complete genome
95638
CM000169.1 Aspergillus fumigatus Af293 chromosome 1, whole genome shotgun sequence
4918979
CM000170.1 Aspergillus fumigatus Af293 chromosome 2, whole genome shotgun sequence
4844472
CM000171.1 Aspergillus fumigatus Af293 chromosome 3, whole genome shotgun sequence
4079167
CM000172.1 Aspergillus fumigatus Af293 chromosome 4, whole genome shotgun sequence
3923705
CM000173.1 Aspergillus fumigatus Af293 chromosome 5, whole genome shotgun sequence
3948441
CM000174.1 Aspergillus fumigatus Af293 chromosome 6, whole genome shotgun sequence
3778736
CM000175.1 Aspergillus fumigatus Af293 chromosome 7, whole genome shotgun sequence
2058334
CP003009.1 Thielavia terrestris NRRL 8126 chromosome 1, complete sequence
10101509
CP003010.1 Thielavia terrestris NRRL 8126 chromosome 2, complete sequence
9477512
CP003011.1 Thielavia terrestris NRRL 8126 chromosome 3, complete sequence
4786945
CP003012.1 Thielavia terrestris NRRL 8126 chromosome 4, complete sequence
4578922
CP003013.1 Thielavia terrestris NRRL 8126 chromosome 5, complete sequence
4396881
CP003014.1 Thielavia terrestris NRRL 8126 chromosome 6, complete sequence
3570487
CH672346.1 Candida albicans WO-1 chromosome 1 supercont1.1 genomic scaffold, whole genome shotgun sequence
3194068
GG670278.1 Candida albicans WO-1 chromosome 1 supercont1.10 genomic scaffold, whole genome shotgun sequence

10847
CH672350.1 Candida albicans WO-1 chromosome 2 supercont1.5 genomic scaffold, whole genome shotgun sequence
1368965
CH672354.1 Candida albicans WO-1 chromosome 2 supercont1.9 genomic scaffold, whole genome shotgun sequence
891553
CM000310.1 Candida albicans WO-1 chromosome 3, whole genome shotgun sequence
1768732
GG670282.1 Candida albicans WO-1 chromosome 4 supercont1.14 genomic scaffold, whole genome shotgun sequence
6607
CH672349.1 Candida albicans WO-1 chromosome 4 supercont1.4 genomic scaffold, whole genome shotgun sequence
1636855
CM000311.1 Candida albicans WO-1 chromosome 5, whole genome shotgun sequence
1198695
CM000312.1 Candida albicans WO-1 chromosome 6, whole genome shotgun sequence
1043947
CM000313.1 Candida albicans WO-1 chromosome 7, whole genome shotgun sequence
958737
CM000309.1 Candida albicans WO-1 chromosome R, whole genome shotgun sequence
2299365
GG670279.1 Candida albicans WO-1 supercont1.11 genomic scaffold, whole genome shotgun sequence
10615
GG670280.1 Candida albicans WO-1 supercont1.12 genomic scaffold, whole genome shotgun sequence
10435
GG670281.1 Candida albicans WO-1 supercont1.13 genomic scaffold, whole genome shotgun sequence
7823
GG670283.1 Candida albicans WO-1 supercont1.15 genomic scaffold, whole genome shotgun sequence
5386
GG670284.1 Candida albicans WO-1 supercont1.16 genomic scaffold, whole genome shotgun sequence
5039
GG670285.1 Candida albicans WO-1 supercont1.17 mitochondrial scaffold, whole genome shotgun sequence
55284
CP017623.1 Candida albicans SC5314 chromosome 1 sequence
3188341
CP017624.1 Candida albicans SC5314 chromosome 2 sequence
2231883
CP017625.1 Candida albicans SC5314 chromosome 3 sequence
1799298
CP017626.1 Candida albicans SC5314 chromosome 4 sequence
1603259
CP017627.1 Candida albicans SC5314 chromosome 5 sequence
1190845
CP017628.1 Candida albicans SC5314 chromosome 6 sequence
1033292
CP017629.1 Candida albicans SC5314 chromosome 7 sequence
949511
CP017630.1 Candida albicans SC5314 chromosome R sequence
2286237
CP025167.1 Candida albicans strain SC5314-GTH12 chromosome 1A
3186784
CP025168.1 Candida albicans strain SC5314-GTH12 chromosome 2A

2231038
CP025169.1 Candida albicans strain SC5314-GTH12 chromosome 3A
1799051
CP025170.1 Candida albicans strain SC5314-GTH12 chromosome 4A
1603040
CP025171.1 Candida albicans strain SC5314-GTH12 chromosome 5A
1190317
CP025172.1 Candida albicans strain SC5314-GTH12 chromosome 6A
1032182
CP025173.1 Candida albicans strain SC5314-GTH12 chromosome 7A
948952
CP025174.1 Candida albicans strain SC5314-GTH12 chromosome RA
2285431
CP025175.1 Candida albicans strain SC5314-GTH12 chromosome 1B
3186276
CP025176.1 Candida albicans strain SC5314-GTH12 chromosome 2B
2231079
CP025177.1 Candida albicans strain SC5314-GTH12 chromosome 3B
1798977
CP025178.1 Candida albicans strain SC5314-GTH12 chromosome 4B
1602746
CP025179.1 Candida albicans strain SC5314-GTH12 chromosome 5B
1190508
CP025180.1 Candida albicans strain SC5314-GTH12 chromosome 6B
1032226
CP025181.1 Candida albicans strain SC5314-GTH12 chromosome 7B
949045
CP025182.1 Candida albicans strain SC5314-GTH12 chromosome RB
2285142
CP025183.1 Candida albicans strain SC5314-GTH12 mitochondrion
40418
CP025150.1 Candida albicans strain SC5314-P0 chromosome 1A
3187190
CP025151.1 Candida albicans strain SC5314-P0 chromosome 2A
2231757
CP025152.1 Candida albicans strain SC5314-P0 chromosome 3A
1799323
CP025153.1 Candida albicans strain SC5314-P0 chromosome 4A
1603391
CP025154.1 Candida albicans strain SC5314-P0 chromosome 5A
1190670
CP025155.1 Candida albicans strain SC5314-P0 chromosome 6A
1032578
CP025156.1 Candida albicans strain SC5314-P0 chromosome 7A
949203
CP025157.1 Candida albicans strain SC5314-P0 chromosome RA
2285906
CP025158.1 Candida albicans strain SC5314-P0 chromosome 1B
3186810
CP025159.1 Candida albicans strain SC5314-P0 chromosome 2B
2231689
CP025160.1 Candida albicans strain SC5314-P0 chromosome 3B
1799405
CP025161.1 Candida albicans strain SC5314-P0 chromosome 4B
1603204
CP025162.1 Candida albicans strain SC5314-P0 chromosome 5B
1190801
CP025163.1 Candida albicans strain SC5314-P0 chromosome 6B
1032709
CP025164.1 Candida albicans strain SC5314-P0 chromosome 7B

949291
CP025165.1 Candida albicans strain SC5314-P0 chromosome RB
2285569
CP025166.1 Candida albicans strain SC5314-P0 mitochondrion
40443
CM001231.1 Magnaporthe oryzae 70-15 chromosome 1, whole genome shotgun sequence
7978604
CM001232.1 Magnaporthe oryzae 70-15 chromosome 2, whole genome shotgun sequence
8319966
CM001233.1 Magnaporthe oryzae 70-15 chromosome 3, whole genome shotgun sequence
6606598
CM001234.1 Magnaporthe oryzae 70-15 chromosome 4, whole genome shotgun sequence
5546968
CM001235.1 Magnaporthe oryzae 70-15 chromosome 5, whole genome shotgun sequence
4490059
CM001236.1 Magnaporthe oryzae 70-15 chromosome 6, whole genome shotgun sequence
4133993
CM001237.1 Magnaporthe oryzae 70-15 chromosome 7, whole genome shotgun sequence
3415785
AE016814.2 Ashbya gossypii ATCC 10895 chromosome I, complete sequence
693414
AE016815.5 Ashbya gossypii ATCC 10895 chromosome II, complete sequence
870771
AE016816.3 Ashbya gossypii ATCC 10895 chromosome III, complete sequence
907494
AE016817.6 Ashbya gossypii ATCC 10895 chromosome IV, complete sequence
1467287
AE016818.2 Ashbya gossypii ATCC 10895 chromosome V, complete sequence
1519140
AE016819.5 Ashbya gossypii ATCC 10895 chromosome VI, complete sequence
1836693
AE016820.4 Ashbya gossypii ATCC 10895 chromosome VII, complete sequence
1800949
AE016821.1 Ashbya gossypii (= Eremothecium gossypii) ATCC 10895 mitochondrion, complete genome
23564
CP002705.1 Ashbya gossypii FDAG1 chromosome I, complete sequence
693310
CP002706.1 Ashbya gossypii FDAG1 chromosome II, complete sequence
874947
CP002707.1 Ashbya gossypii FDAG1 chromosome III, complete sequence
911446
CP002708.1 Ashbya gossypii FDAG1 chromosome IV, complete sequence
1465717
CP002709.1 Ashbya gossypii FDAG1 chromosome V, complete sequence
1527143
CP002710.1 Ashbya gossypii FDAG1 chromosome VI, complete sequence
1837132
CP002711.1 Ashbya gossypii FDAG1 chromosome VII, complete sequence
1823962
CP002712.1 Ashbya gossypii FDAG1 mitochondrion, complete sequence
23497
CM001563.1 Saccharomyces arboricola H-6 chromosome I, whole genome shotgun sequence
194368
CM001564.1 Saccharomyces arboricola H-6 chromosome II, whole genome shotgun sequence
782499
CM001565.1 Saccharomyces arboricola H-6 chromosome III, whole genome shotgun sequence
298424
CM001566.1 Saccharomyces arboricola H-6 chromosome IV, whole genome shotgun sequence
1141293

CM001567.1 Saccharomyces arboricola H-6 chromosome V, whole genome shotgun sequence
531626
CM001568.1 Saccharomyces arboricola H-6 chromosome VI, whole genome shotgun sequence
272605
CM001569.1 Saccharomyces arboricola H-6 chromosome VII, whole genome shotgun sequence
1053431
CM001570.1 Saccharomyces arboricola H-6 chromosome VIII, whole genome shotgun sequence
527949
CM001571.1 Saccharomyces arboricola H-6 chromosome IX, whole genome shotgun sequence
407361
CM001572.1 Saccharomyces arboricola H-6 chromosome X, whole genome shotgun sequence
721635
CM001573.1 Saccharomyces arboricola H-6 chromosome XI, whole genome shotgun sequence
650640
CM001574.1 Saccharomyces arboricola H-6 chromosome XII, whole genome shotgun sequence
984206
CM001575.1 Saccharomyces arboricola H-6 chromosome XIII, whole genome shotgun sequence
1246548
CM001576.1 Saccharomyces arboricola H-6 chromosome XIV, whole genome shotgun sequence
771513
CM001577.1 Saccharomyces arboricola H-6 chromosome XV, whole genome shotgun sequence
1023324
CM001578.1 Saccharomyces arboricola H-6 chromosome XVI, whole genome shotgun sequence
879294
CM000687.1 Saccharomyces kluyveri NRRL Y-12651 chromosome A, whole genome shotgun sequence
951467
CM000688.1 Saccharomyces kluyveri NRRL Y-12651 chromosome B, whole genome shotgun sequence
1119477
CM000689.1 Saccharomyces kluyveri NRRL Y-12651 chromosome C, whole genome shotgun sequence
1252455
CM000690.1 Saccharomyces kluyveri NRRL Y-12651 chromosome D, whole genome shotgun sequence
1289280
CM000691.1 Saccharomyces kluyveri NRRL Y-12651 chromosome E, whole genome shotgun sequence
1295560
CM000692.1 Saccharomyces kluyveri NRRL Y-12651 chromosome F, whole genome shotgun sequence
1385275
CM000693.1 Saccharomyces kluyveri NRRL Y-12651 chromosome G, whole genome shotgun sequence
1737261
CM000694.1 Saccharomyces kluyveri NRRL Y-12651 chromosome H, whole genome shotgun sequence
2314951
CP001942.1 Encephalitozoon intestinalis ATCC 50506 chromosome I, complete sequence
160332
CP001943.1 Encephalitozoon intestinalis ATCC 50506 chromosome II, complete sequence
175776
CP001944.1 Encephalitozoon intestinalis ATCC 50506 chromosome III, complete sequence
176815
CP001945.1 Encephalitozoon intestinalis ATCC 50506 chromosome IV, complete sequence
193740
CP001946.1 Encephalitozoon intestinalis ATCC 50506 chromosome V, complete sequence
196642
CP001947.1 Encephalitozoon intestinalis ATCC 50506 chromosome VI, complete sequence
198217

CP001948.1 Encephalitozoon intestinalis ATCC 50506 chromosome VII, complete sequence
205935
CP001949.1 Encephalitozoon intestinalis ATCC 50506 chromosome VIII, complete sequence
204910
CP001950.1 Encephalitozoon intestinalis ATCC 50506 chromosome IX sequence
233397
CP001951.1 Encephalitozoon intestinalis ATCC 50506 chromosome X, complete sequence
234890
CP001952.1 Encephalitozoon intestinalis ATCC 50506 chromosome XI, complete sequence
236244
CP010913.1 Sporisorium scitamineum strain SSC39 chromosome 1, complete sequence
2009762
CP010914.1 Sporisorium scitamineum strain SSC39 chromosome 2, complete sequence
1671606
CP010915.1 Sporisorium scitamineum strain SSC39 chromosome 3, complete sequence
1566833
CP010916.1 Sporisorium scitamineum strain SSC39 chromosome 4, complete sequence
1271980
CP010917.1 Sporisorium scitamineum strain SSC39 chromosome 5, complete sequence
1055339
CP010918.1 Sporisorium scitamineum strain SSC39 chromosome 6, complete sequence
1032954
CP010919.1 Sporisorium scitamineum strain SSC39 chromosome 7, complete sequence
938453
CP010920.1 Sporisorium scitamineum strain SSC39 chromosome 8, complete sequence
837966
CP010921.1 Sporisorium scitamineum strain SSC39 chromosome 9, complete sequence
808176
CP010922.1 Sporisorium scitamineum strain SSC39 chromosome 10, complete sequence
875830
CP010923.1 Sporisorium scitamineum strain SSC39 chromosome 11, complete sequence
790822
CP010924.1 Sporisorium scitamineum strain SSC39 chromosome 12, complete sequence
716888
CP010925.1 Sporisorium scitamineum strain SSC39 chromosome 13, complete sequence
681008
CP010926.1 Sporisorium scitamineum strain SSC39 chromosome 14, complete sequence
652314
CP010927.1 Sporisorium scitamineum strain SSC39 chromosome 15, complete sequence
634981
CP010928.1 Sporisorium scitamineum strain SSC39 chromosome 16, complete sequence
653278
CP010929.1 Sporisorium scitamineum strain SSC39 chromosome 17, complete sequence
605135
CP010930.1 Sporisorium scitamineum strain SSC39 chromosome 18, complete sequence
598089
CP010931.1 Sporisorium scitamineum strain SSC39 chromosome 19, complete sequence
580969
CP010932.1 Sporisorium scitamineum strain SSC39 chromosome 20, complete sequence
573042
CP010933.1 Sporisorium scitamineum strain SSC39 chromosome 21, complete sequence
539152
CP010934.1 Sporisorium scitamineum strain SSC39 chromosome 22, complete sequence
475902
CP010935.1 Sporisorium scitamineum strain SSC39 chromosome 23, complete sequence
140422
CP010936.1 Sporisorium scitamineum strain SSC39 chromosome 24 sequence
87293
CP010937.1 Sporisorium scitamineum strain SSC39 chromosome 25 sequence
82225

CP010938.1 Sporisorium scitamineum strain SSC39 chromosome 26 sequence
99152
CP010939.1 Sporisorium scitamineum strain SSC39 mitochondrion, complete sequence
88018
>NC_031675.1 Fusarium verticillioides 7600 chromosome 1, whole genome shotgun sequence
6219215
