# Supplementary figures and images for: HumanMycobiomeScan: a new bioinformatics tool for the characterization of the fungal fraction in metagenomic samples
Source: BMC Genomics. 2019 Jun 15;20:496. doi: 10.1186/s12864-019-5883-y (PMC6570844; doi:10.1186/s12864-019-5883-y)

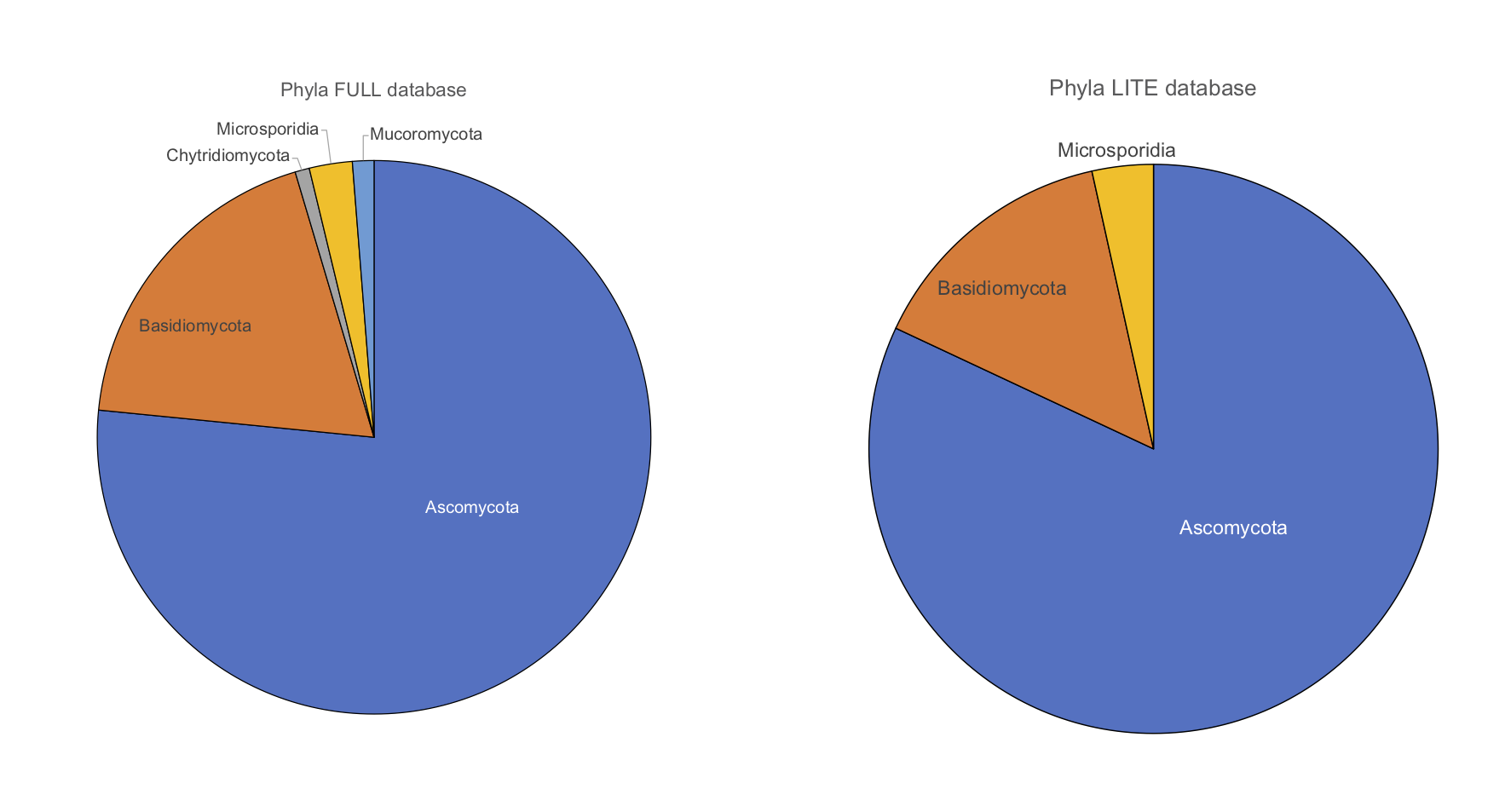

Supplement: Supplementary file 2 — Genomes in the two databases are represented as pie charts color-coded by phylum assignment. (TIFF 158 kb) [file 12864_2019_5883_MOESM2_ESM.tiff]
