# Supplementary material for: HumanMycobiomeScan: a new bioinformatics tool for the characterization of the fungal fraction in metagenomic samples
Source: BMC Genomics. 2019 Jun 15;20:496. doi: 10.1186/s12864-019-5883-y (PMC6570844; doi:10.1186/s12864-019-5883-y)
Supplement: Supplementary file 3 — Genomes used to generate the synthetic meta-communities used in the HumanMycobiomeScan validation process. (PDF 22 kb) [file 12864_2019_5883_MOESM3_ESM.pdf]

Mock 1

NC_007197.1	Aspergillus fumigatus
NC_016459.1	Thielavia terrestris
NC_014945.1	Cryptococcus gattii
NC_026755.1	Cryptococcus neoformans
CM000312.1	Candida albicans
NC_009044.1	Scheffersomyces stipitis
NC_017844.1	Magnaporthe oryzae
CM000578.1	Fusarium verticillioides
FR839629.1	Komagataella phaffii
NC_005788.4	Ashbya gossypii
HE978316.1	Kazachstania naganishii
CM001570.1	Saccharomyces arboricola
NC_001136.10	Saccharomyces cerevisiae
CM003578.1	Saccharomyces eubayanus
CM000692.1	Saccharomyces kluyveri
NC_020189.1	Tetrapisispora blattae
NC_026505.1	Neurospora crassa
NC_014422.1	Encephalitozoon intestinalis
NC_024901.1	Encephalitozoon romaleae
CP010929.1	Sporisorium scitamineum
NC_001531.1	Human papillomoavirus type 5
NC_000913.3	Escherichia coli
FP929046.1	Faecalibacterium prausnitzii
NC_002737.2	Streptococcus pyogenes
AE006468.1	Salmonella enterica
NC_014833.1	Ruminococcus albus
NOS2homo	NOS2 homo sapiens

Mock 2

NC_003421.2	Schizosaccharomyces;pombe
NC_026506.1	Neurospora;crassa
NC_003232.1	Encephalitozoon;cuniculi
NC_026476.1	Fusarium;graminearum
NC_026480.1	Ustilago;maydis
NC_016495.1	Naumovozyma;castellii
NC_006039.1	Kluyveromyces;lactis
NC_006071.1	Yarrowia;lipolytica
NC_006048.2	Debaryomyces;hansenii
NC_013083.1	Kluyveromyces;thermotolerans
NC_020215.1	Millerozyma;farinosa
CP014586.1	Komagataella;pastoris
NC_012861.1	Candida;dubliniensis
CM000597.1	Fusarium;oxysporum
NC_016459.1	Thielavia;terrestris
FQ311439.1	Sporisorium;reilianum
AP012219.1	Kluyveromyces;marxianus
NC_018475.1	Encephalitozoon;hellem
CM003109.1	Valsa;mali
FR839631.1	Pichia;pastoris
NC_006347.1	Bacteroides fragilis
NC_022124.1	Prevotella sp.
NC_017960.1	Enterococcus faecium
NC_007795.1	Staphylococcus aureus

NC_015738.1 Eggerthella sp
NC_000902.1 Enterobacteria phage
NC_000007.14 region EGFR

Mock 3
NC_007197.1 Aspergillus fumigatus
NC_016459.1 Thielavia terrestris
NC_014945.1 Cryptococcus gattii
NC_026755.1 Cryptococcus neoformans
CM000312.1 Candida albicans
NC_009044.1 Scheffersomyces stipitis
NC_017844.1 Magnaporthe oryzae
CM000578.1 Fusarium verticillioides
FR839629.1 Komagataella phaffii
NC_005788.4 Ashbya gossypii
HE978316.1 Kazachstania naganishii
CM001570.1 Saccharomyces arboricola
NC_001136.10 Saccharomyces cerevisiae
CM003578.1 Saccharomyces eubayanus
CM000692.1 Saccharomyces kluyveri
NC_020189.1 Tetrapisispora blattae
NC_026505.1 Neurospora crassa
NC_014422.1 Encephalitozoon intestinalis
NC_024901.1 Encephalitozoon romaleae
CP010929.1 Sporisorium scitamineum
NC_001531.1 Human papillomoavirus type 5
NC_000913.3 Escherichia coli
FP929046.1 Faecalibacterium prausnitzii
NC_002737.2 Streptococcus pyogenes
AE006468.1 Salmonella enterica
NC_014833.1 Ruminococcus albus
NC_000007.14 region EGFR human

Mock 4
NC_003421.2 Schizosaccharomyces;pombe
NC_026506.1 Neurospora;crassa
NC_003232.1 Encephalitozoon;cuniculi
NC_026476.1 Fusarium;graminearum
NC_026480.1 Ustilago;maydis
NC_016495.1 Naumovozyma;castellii
NC_006039.1 Kluyveromyces;lactis
NC_006071.1 Yarrowia;lipolytica
NC_006048.2 Debaryomyces;hansenii
NC_013083.1 Kluyveromyces;thermotolerans
NC_020215.1 Millerozyma;farinosa
CP014586.1 Komagataella;pastoris
NC_012861.1 Candida;dubliniensis
CM000597.1 Fusarium;oxysporum
NC_016459.1 Thielavia;terrestris
FQ311439.1 Sporisorium;reilianum
AP012219.1 Kluyveromyces;marxianus
NC_018475.1 Encephalitozoon;hellem
CM003109.1 Valsa;mali
FR839631.1 Pichia;pastoris

NC_006347.1 Bacteroides fragilis
NC_022124.1 Prevotella sp.
NC_017960.1 Enterococcus faecium
NC_007795.1 Staphylococcus aureus
NC_015738.1 Eggerthella sp
NC_000902.1 Enterobacteria phage
NC_000007.14 region EGFR

Mock 5
CM002798.1 Penicillium chrysogenum
NC_026501.1 Neurospora crassa
NC_009614.1 Bacteroides vulgatus
BN001308.1 Aspergillus Nidulans
NC_006049.2 Debaryomyces hansenii
NC_020217.1 Millerozyma farinosa
CM002778.1 Yarrowia lipolytica
CM004459.1 Glomerellaceae Colletotrichum higginsianum
NC_000019.10 glucose-6-phosphate isomerase human
NC_007455.1 Human bocavirus
CP010983.1 Verticillium dahliae
NZ_ACTW00000000.1 Lachnospiraceae bacterium 7_1_58FAA
HF679034.1 Fusarium fujikuroi
NC_026474.1 Fusarium graminearum
CM004558.1 Fusarium pseudograminearum
NC_009615.1 Parabacteroides distasonis
NZ_AQHV00000000.1 Parabacteroides goldsteinii
DG000067.1 Cyberlindnera jadinii
NC_027863.1 Ogataea parapolymorpha
NZ_ACFY00000000.1 Roseburia inulinivorans
NC_018941.1 Kazachstania africana
CM007073.1 Saccharomyces cerevisiae
CP011784.1 Torulaspora delbrueckii
NC_012994.1 Zygosaccharomyces rouxii
NC_003421.2 Schizosaccharomyces pombe
CP009805.1 Botrytis cinerea
NC_003237.1 Encephalitozoon cuniculi
